# Cellodextrin phosphorylase from *Ruminiclostridium thermocellum*: X-ray crystal structure and substrate specificity analysis

**DOI:** 10.1016/j.carres.2017.07.005

**Published:** 2017-11-08

**Authors:** Ellis C. O'Neill, Giulia Pergolizzi, Clare E.M. Stevenson, David M. Lawson, Sergey A. Nepogodiev, Robert A. Field

**Affiliations:** Department of Biological Chemistry, John Innes Centre, Norwich Research Park, Norwich NR4 7UH, UK

**Keywords:** Cellodextrin phosphorylase, Glucosamine 1-phosphate, X-ray crystal structure

## Abstract

The GH94 glycoside hydrolase cellodextrin phosphorylase (CDP, EC 2.4.1.49) produces cellodextrin oligomers from short β-1→4-glucans and α-D-glucose 1-phosphate. Compared to cellobiose phosphorylase (CBP), which produces cellobiose from glucose and α-D-glucose 1-phosphate, CDP is biochemically less well characterised. Herein, we investigate the donor and acceptor substrate specificity of recombinant CDP from *Ruminiclostridium thermocellum* and we isolate and characterise a glucosamine addition product to the cellobiose acceptor with the non-natural donor α-D-glucosamine 1-phosphate. In addition, we report the first X-ray crystal structure of CDP, along with comparison to the available structures from CBPs and other closely related enzymes, which contributes to understanding of the key structural features necessary to discriminate between monosaccharide (CBP) and oligosaccharide (CDP) acceptor substrates.

## Highlights

• Screening of CDP activity against a variety of donors and acceptors.
• GlcN-1-P acts as CDP donor and generates a single turnover product.
• Xyl-1-P and Man-1-P are poor donors but competitive inhibitors of CDP.
• Oligosaccharides with GlcN and Xyl at the non-reducing end are good CDP acceptors.
• The apo structure of CDP and its complex with cellotetraose and Pi have been resolved.

## Graphical abstract

**Figure:**
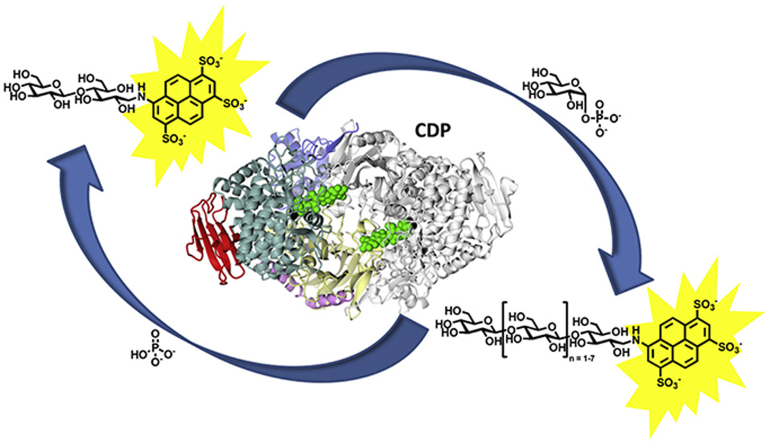

## 1 Introduction

β-1→4-Linked glucan polysaccharides are widespread in nature, where they are principally involved in structural roles [1]. The most common example, cellulose, is the main component of primary and secondary plant cell walls [1], [2]; however, cellulose is also found in bacteria [3], algae [4], and oomycetes [5]. It is composed of extensive, unbranched chains of β-1→4-linked D-glucose (Glc), where the residues alternate orientation such that the overall molecular structure may be considered as a repeating polymer of cellobiose blocks [1], [2]. Extensive *intra*/*inter* chain hydrogen bonds can be generated by β-1→4-glucans, which support the arrangement of glucan chains in microfibrils with highly ordered crystalline regions [1], [2], [6], [7], conferring upon them stiffness and high resistance to thermal and enzymatic degradation. Among other common β-1→4-glucans are the xyloglucans, which span cellulose microfibrils, generating a 3D network in the plant cell wall [8], [9]. Its unique physical-chemical properties make cellulose, its derivatives and analogues, suitable for a wide variety of applications, spanning from paper products, to textiles, food thickeners and stabilizers, to composite materials and hydrogels for sensors development, medical, electronic and pharmaceutical applications [2], [6], [10], [11], [12]. However, routine access to pure cellulose at scale is challenging due to its association with hemicellulose and lignin in plant materials [13]. In contrast, bacterial cellulose is synthesised in a much purer form, albeit with a different crystalline structure [3], [14].

The prospect of using cellulose-producing enzymes *in vitro* as an eco-friendly alternative to obtain pure cellulose is potentially attractive, although the natural biosynthetic machinery comprises a very complex, multi-protein *trans*-membrane system [15]. As an alternative, cellulose-like material has been synthesised by Kobayashi and co-workers using a hydrolytic cellulase in a reverse synthetic reaction with β-cellobiosyl fluoride as substrate in a mixture of acetonitrile/buffer [16]. Recently, phosphorylases have received attention as catalysts for glycoside synthesis [17], [18], including the production of α- and β-1→4-linked glucans. For instance, the plant α-1→4-glucan phosphorylase PHS2 has been used to generate starch-like materials [19], while cellodextrin phosphorylase (CDP) from *Ruminiclostridium thermocellum* (formerly *Clostridium thermocellum*) [20], [21], [22] has been shown to be a suitable enzyme for cello-oligosaccharide and cellulose production [23], [24] using short cellodextrins and α-D-glucose 1-phosphate (Glc-1-P) as substrates. The latter reactions are simple, eco-friendly and produce cellulose oligomers with different degrees of polymerization (DP) depending on the concentration of the substrates [23]. Hiraishi *et al.* described the synthesis of crystalline cellulose-like material with an average of ∼ DP 9 using high concentration of glucose as CDP acceptor [24].

CDP (EC 2.4.1.49) belongs to the glycoside hydrolase family GH94 in the Carbohydrate Active Enzyme (CAZY) database (URL: http://www.cazy.org/) [25], along with cellobiose phosphorylase (CBP), which has been extensively characterised from a variety of sources [26], [27], [28], [29]. For a summary of established CBP acceptor and donor specificity, see Tables S1 and S2. Less comprehensive studies have been conducted on CDP activity and specificity; it has been used to synthesise a variety of cellulose derivatives, assessing its permissiveness toward acceptors (Table S1), but less information is available about its donor specificity [30], [31] (Table S2) and no X-ray crystal structure is available for this enzyme. However, recombinant CDP can be produced in high yield in *E. coli* [32], [33] and it is stable up to 60 °C with highest activity at pH 7.5 [33], making it suitable for process development. Protein sequence alignment of *R. thermocellum* CDP with CBP from the same organism shows that the two enzymes share only ∼17% identity. To fully understand how CDP and CBP discriminate between glucose and cello-oligosaccharide acceptor substrates a structural comparison of CDP and CBP would be informative.

Herein, we report studies that investigate the donor and acceptor specificity of recombinant *R. thermocellum* CDP. Where low or no turnover was observed, additional inhibition experiments were performed to probe the interaction between the sugar 1-phosphates or oligosaccharides and the enzyme. In addition, X-ray crystallography was used to characterise the structure of CDP and compare it to known structures of CBP.

## 2 Results and discussion

### 2.1 Protein expression and activity assay

The *cdp* gene from *Ruminiclostridium thermocellum* YM4 strain (GenBank accession number BAB71818) was synthesised with codon optimization for expression in *E. coli* and sub-cloned into pET15b, which inserts a hexahistidine tag behind a thrombin cleavage site at the N-terminus of the protein (see supplementary information for nucleotide and amino acids sequences). Protein was expressed and purified using a combination of nickel affinity and gel filtration chromatography (Supplementary Figs. S1–S2). The resulting CDP protein consists of 1009 amino acids, with a molecular weight of 114.364 KDa per monomer, in accordance with the GF elution profile. A yield of ∼10 mg of purified CDP per litre of culture was obtained, which was concentrated to 40 mg/ml and stored at −80 °C until required.

**Fig. 1:**
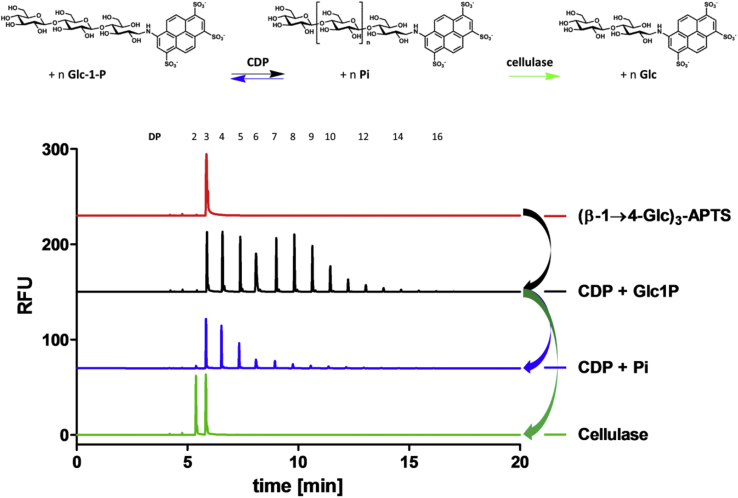
**Carbohydrate electrophoresis of CDP-synthesised oligomers and reaction scheme.** The activity of the phosphorylase was confirmed by assaying the ability of CDP (1 μg) to transfer Glc from Glc-1-P (disodium salt, 50 mM) on to (β-1→4-Glc)_3_-APTS (5 mM) in 100 μl HEPES buffer (50 mM, pH 7.6) (all concentrations are final concentrations). After 2 h at 40 °C, the reactions were terminated by heating to 95 °C in a boiling water bath for 5 min and centrifuging at 16,000 g for 5 min. 20 μl of this synthetic reaction were further probed by degradation with *endo*-cellulase (0.7 U) from *T. longibrachiatum* (Megazyme) in HEPES buffer (50 mM, pH 7.6) for 2 h at 40 °C, or CDP (5 μg) in 1 × PBS buffer (0.01 M phosphate buffer, 3 mM potassium chloride and 140 mM sodium chloride, pH 7.4), for 1 h at 40 °C, before again heating to 95 °C and centrifuging as before.

The ability of CDP to synthesise and phosphorolyse cello-oligosaccharides was monitored using capillary electrophoresis with laser-induced fluorescence detection to assess the degree of polymerization (Fig. 1) [34], [35]. Cello-oligosaccharides were labelled by reductive amination with the fluorophore 8-amino-1,3,6-pyrenetrisulfonic acid (APTS) [34], [35]. CDP was able to extend APTS-labelled cellotriose, (β-1→4-Glc)_3_-APTS (Fig. 1, red), to oligomers up to DP 16 (Fig. 1, black) by transferring glucose from Glc-1-P on to the acceptor, although most of the synthesised material was insoluble and removed during the sample preparation. Indeed, cello-oligosaccharides beyond ∼ DP 9 are known to have limited aqueous solubility [24]. CDP could also phosphorolyse the CDP-synthesised APTS-labelled oligomers, reducing the chain length up to (β-1→4-Glc)_3_-APTS (Fig. 1, blue). *Endo*-cellulase from *Trichoderma longibrachiatum* was able to hydrolyse the CDP-synthesised APTS-labelled oligomers (Fig. 1, black) to (β-1→4-Glc)_3_-APTS and (β-1→4-Glc)_2_-APTS (Fig. 1, green), confirming the CDP-generated material was indeed β-1→4-linked glucan.

### 2.2 Donor specificity of CDP

**Fig. 2:**
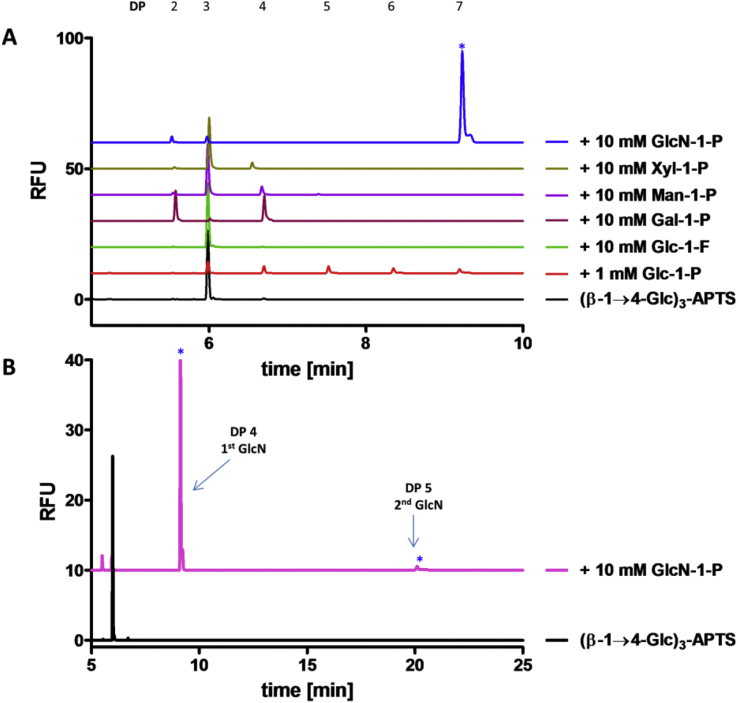
**Donor specificity of CDP analysed using carbohydrate electrophoresis. A. Extension of (β-1→4-Glc)**_**3**_**-APTS (black) with various sugar 1-P donors (15 min run). B. Extension of (β-1→4-Glc)**_**3**_**-APTS (black) with GlcN-1-P (pink, 30 min run).** Assays were carried out using CDP (5 μg/ml) at 40 °C with (β-1→4-Glc)_3_-APTS acceptor (2 μM), Glc-1-P (disodium salt, 1 mM), or other sugar 1-P donors (10 mM, Gal-1-P: dipotassium salt pentahydrate; Man-1-P: disodium salt; Xyl-1-P: bis(cyclohexylammonium) salt) in HEPES buffer (50 mM, pH 7.5) (all concentrations are final concentrations), followed by heating to 95 °C in a boiling water bath for 5 min and centrifuging at 16,000 g for 5 min. CE was performed under standard conditions. The symbol * highlights that the DP of marked peaks does not correspond to the DP of all other neutral species peaks. (For interpretation of the references to colour in this figure legend, the reader is referred to the web version of this article.)

In order to determine the capability of CDP for the general synthesis of β-1→4-linked oligomers not based on glucose, the transfer of other sugars onto (β-1→4-Glc)_3_-APTS, an efficient acceptor (Fig. 2A, black), was monitored using CE. There was little material visible in the reaction using Glc-1-P (Fig. 2A, red), as the longer oligomeric products formed were insoluble and so removed during sample preparation for the CE analysis. Evaluation of fluoride as a reactive alternative leaving group to phosphate established that synthetic α-D-glucose 1-fluoride (Glc-1-F) was not an effective donor substrate for CDP (Fig. 2A, green), at least under the assay conditions used for CE analysis. However, we note that Nakai and co-workers showed CDP-catalysed addition of Glc from Glc-1-F onto cellobiose in 68% yield despite working with CDP from the same organism [36].

A series of non-cognate sugar 1-phosphate donors – α-D-galactose 1-phosphate (Gal-1-P), α-D-mannose 1-phosphate (Man-1-P), α-D-xylose 1-phosphate (Xyl-1-P) and α-D-glucosamine 1-phosphate (GlcN-1-P) – were also assessed as prospective CDP substrates, with (β-1→4-Glc)_3_-APTS as the acceptor. In the presence of Gal-1-P (Fig. 2A, magenta), the formation of a peak with DP 2 and a peak with DP 4 may be accounted for addition of a single Gal onto the acceptor and concurrent phosphorolysis of remaining (β-1→4-Glc)_3_-APTS acceptor to (β-1→4-Glc)_2_-APTS. Indeed, it has been previously reported that Gal-1-P is a donor for CDP from *C. stercorarium*, even if the *k*_cat_/*K*_M_ for Gal-1-P with CDP is ∼1% of that for Glc-1-P [31]. In the reaction with Man-1-P (Fig. 2A, purple), a small additional peak may correspond to the addition of one mannose residue or it may be accounted for acceptor disproportionation [30]; this could be due to any residual phosphate present in the reaction mixture, which would allow phosphorolysis of the APTS-labelled acceptor and utilisation of the resulting Glc-1-P for transfer of Glc onto another molecule of (β-1→4-Glc)_3_-APTS acceptor. Where Xyl-1-P was used (Fig. 2A, olive), a very limited turnover was evident, with possible xylose transfer product formation suggested by a new peak that appears at a slightly faster retention time than the corresponding (β-1→4-Glc)_4_-APTS. Shintate and co-workers previously demonstrated that CDP from *R. thermocellum* can utilise Xyl-1-P as donor, although its activity is only a few % of that of Glc-1-P [30]. In stark contrast, the reaction with GlcN-1-P showed almost complete conversion of the acceptor substrate to a single much later-running peak on the electropherogram (Fig. 2A, blue). This could be the product of a single turnover arising from the addition of one glucosamine (GlcN) residue which, at the pH of the running buffer (pH 4.75), is protonated and could account for the long retention time. In more extended electrophoresis runs, another weak peak is evident, suggesting the possible addition of a further GlcN unit (Fig. 2B, pink).

### 2.3 Isolation and characterization of the GlcN addition product, (β-1→4-GlcN(Glc)_2_)

**Fig. 3:**
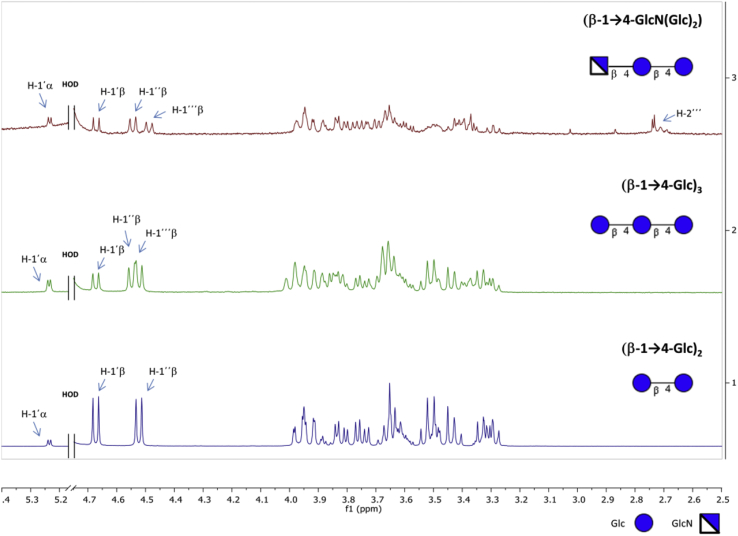
^**1**^**H-NMR of isolated (β-1→4-GlcN(Glc)**_**2**_**) product in comparison with β-1→4-glucan standards in D**_**2**_**O.** Cellobiose, (β-1→4-Glc)_2_ (blue); cellotriose, (β-1→4-Glc)_3_ (green); (β-1→4-GlcN(Glc)_2_) (red). The spectra were recorded at rt and referenced to HOD (δ_H_ 4.79). HOD signal is omitted for clarity. The symbols ′, ′′ and ′′′ denote the first, second and third glycosyl residue from the reducing end, respectively. (For interpretation of the references to colour in this figure legend, the reader is referred to the web version of this article.)

In order to confirm the identity of any late running product observed by CE, reactions with unlabelled acceptor were scaled-up to monitor them by thin layer chromatography (TLC) and to isolate the products for further NMR and MS characterization. In particular, we focused our attention on the reaction of CDP with GlcN-1-P as donor and (β-1→4-Glc)_2_ as good CDP acceptor but only a very weak substrate for interfering phosphorolysis. This reaction generated mainly a single product in accordance with what was observed by CE (Fig. 2A, blue and 2B, pink). As far as we are aware, there is no precedent for CDP or CBP from any organism using GlcN-1-P as a donor substrate. The crude reaction mixture was fractionated by strong cation exchange chromatography to isolate any positively charged product, which was then eluted with a 0.05 M ammonium bicarbonate buffer (pH 9.4). The collected fractions were analysed by MALDI-ToF and ESI-MS and showed the presence of a trisaccharide containing GlcN instead of Glc (−1 m/z unit difference, Supplementary Figs. S3A and S3B) as the main product; however, traces of multiple Glc addition products were observed in variable amounts from batch to batch, presumably arising from a limited phosphorolysis of (β-1→4-Glc)_2_ generating Glc-1-P *in situ*. In addition, ^1^H-NMR spectroscopy data for the product in D_2_O (pD 7) in comparison with cellobiose and cellotriose standards confirmed the attachment of a GlcN unit to cellobiose due to the presence of a third anomeric proton and to the characteristic signal of H-2′′′ at 2.72 ppm [37] (Fig. 3, Table S3). 2D-COSY and 2D-HSQC spectra (Supplementary Table S3, Figs. S4 and S5), in comparison with those for cellotriose, supported the identity of the main product as (β-1→4-GlcN(Glc)_2_).

### 2.4 Competition experiment with mannose 1-phosphate donor

In order to assess the binding of donor analogues that are extremely poor substrates for CDP, we screened them as prospective inhibitors of the natural CDP substrates. Competition assays were performed to understand whether the lack of efficient activity for sugar 1-phosphate donors different from Glc-1-P is caused by failure to bind them or inefficient catalysis. For Gal-1-P reactions with CDP from *C. stercorarium*, it has been shown that *K*_M_ is increased ∼12 fold and *k*_cat_ reduced ∼10 fold with respect to the corresponding reaction with Glc-1-P, for instance [31]. Xyl-1-P is known to be a poor donor substrate for CDP compared to Glc-1-P [30]; nevertheless, Shintate and co-workers were able to enzymatically synthesise a series of β-1→4-linked-heterooligosaccharides containing Xyl and Glc [30].

**Fig. 4:**
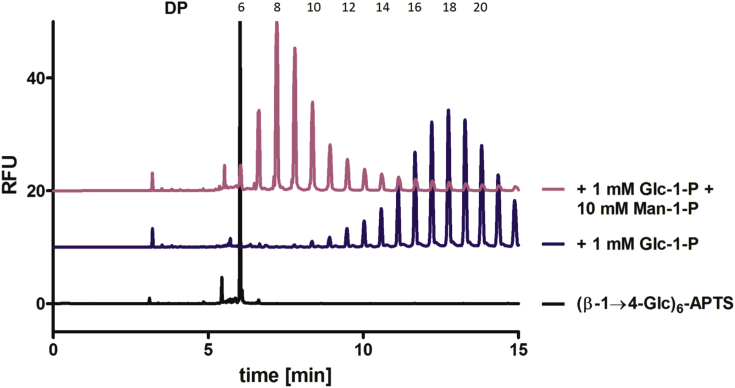
**Man-1-P binding inhibits CDP activity.** CDP oligomerization of (β-1→4-Glc)_6_-APTS (black) with Glc-1-P (blue) in the presence of Man-1-P (pink). Assays were carried out using CDP (5 μg/ml) at 40 °C with (β-1→4-Glc)_6_-APTS (2 μM), Glc-1-P (disodium salt, 1 mM) in HEPES buffer (50 mM, pH 7.5) adding Man-1-P (disodium salt, 10 mM) (all concentrations are final concentrations), followed by heating to 95 °C in a boiling water bath for 5 min and centrifuging at 16,000 g for 5 min. CE was performed under standard conditions. (For interpretation of the references to colour in this figure legend, the reader is referred to the web version of this article.)

Reaction mixtures containing fixed concentrations of Glc-1-P (1 mM) and the acceptor (β-1→4-Glc)_6_-APTS (2 μM) had mannose 1-phosphate (10 mM) added to assess the impact on glucan polymerization (Fig. 4). Man-1-P caused a great decrease in glucan oligomer length (Fig. 4, pink), suggesting binding to the active site although we were unable to demonstrate CDP-catalysed mannose transfer from Man-1-P.

### 2.5 Acceptor specificity of CDP

#### 2.5.1 Acceptor specificity for β-1→4-glucans

The length specificity of CDP for unlabelled reducing glycan acceptors was determined by measurement of phosphate release from 10 mM Glc-1-P over a range of acceptor concentrations (Supplementary Fig. S6). Using this assay, the capability of CDP to use cello-oligosaccharides as acceptors was assessed (Table 1). Glucose was found to be a poor acceptor [24], whilst the monosaccharide derivative phenyl β-d-glucopyranoside was a better acceptor; the latter effect could be due to its fixed anomeric configuration and/or to aglycone aryl groups commonly known for binding well into sugar binding sites. According to the literature, the cello-oligosaccharides displayed a decreasing *K*_M_^app^ up to DP 5 (from 2.6 mM down to 0.36 mM) [24], indicating there are stabilising interactions with the sugars remote from the catalytic site. However, cellohexaose has a higher *K*_M_^app^ (1.9 mM), which may reflect its modest aqueous solubility. The *k*_*cat*_ values follow a similar pattern with respect to acceptor chain length, dropping from DP 2–5 and increasing again at DP 6. The net result is that for acceptors from DP 2–6, the catalytic efficiency, as judged by *k*_cat_/*K*_M_^app^, is consistent within a factor of 2.

**Table 1:** **Acceptor length specificity for CDP.** The length specificity of CDP for reducing glycan acceptors was determined by measurement of phosphate release from 10 mM Glc-1-P (disodium salt) over a range of acceptor concentrations. Michaelis-Menten graphs were plotted using Grafit (Erithacus Software Ltd) (Supplementary Fig. S6).

Acceptor	*K*_M_^app^ (mM)	*k*_cat_ (1/s)	*k*_cat_*/K*_M_^app^ (1/mM/s)
Glc	n.d.	n.d.	n.d.
phenyl β-d-glucopyranoside	24 ± 13	15 ± 6.3	0.63
(β-1→4-Glc)_2_	2.6 ± 0.18	17 ± 0.50	6.5
(β-1→4-Glc)_3_	0.68 ± 0.076	9.5 ± 0.35	14
(β-1→4-Glc)_4_	0.54 ± 0.13	5.0 ± 0.25	9.3
(β-1→4-Glc)_5_	0.36 ± 0.076	4.3 ± 0.47	12
(β-1→4-Glc)_6_	1.9 ± 0.76	7.6 ± 1.8	4.0

n.d. = not determined.

The ability of CDP to use APTS labelled (β-1→4-Glc)_2_, (β-1→4-Glc)_3_ and (β-1→4-Glc)_6_ was monitored over time using CE (Supplementary Fig. S7). (β-1→4-Glc)_2_-APTS is a very poor CDP acceptor, as shown by its slow consumption by CDP [24]; when a single glucose residue is added, the formed (β-1→4-Glc)_3_-APTS is a much better acceptor and it is rapidly oligomerised to insoluble cello-oligosaccharides (Supplementary Fig. S7A). (β-1→4-Glc)_3_-APTS is a much better acceptor than (β-1→4-Glc)_2_-APTS, being completely consumed within 10 min (Supplementary Fig. S7B). (β-1→4-Glc)_6_-APTS is also a good acceptor for CDP, forming longer oligomers rapidly (Supplementary Fig. S7C). This can be seen in the CE as a non-processive reaction, with all oligomers extending in parallel until a combination of chain length and time results in precipitation.

#### 2.5.2 Acceptor specificity for β-1→4-glycans

**Fig. 5:**
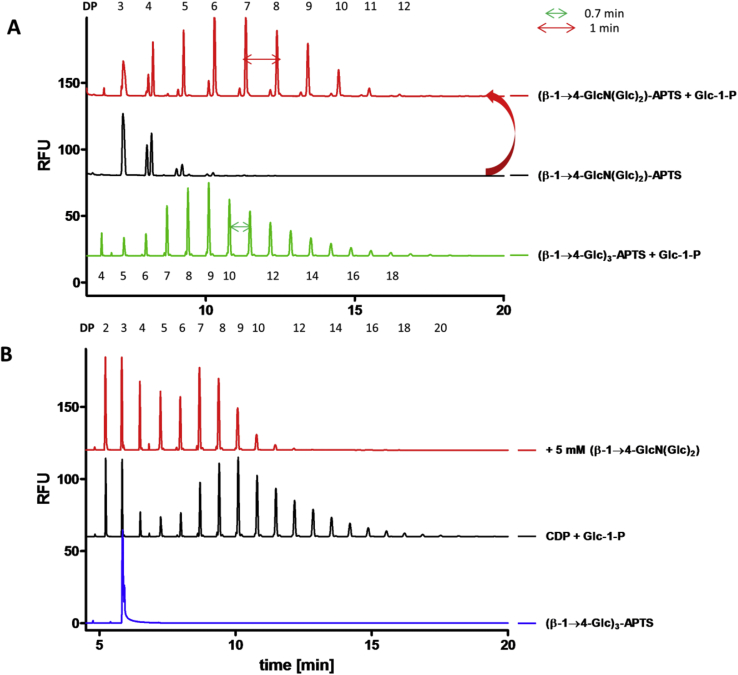
**(β-1→4-GlcN(Glc)**_**2**_**) as CDP acceptor: CE analysis. A. CDP oligomerization of (β-1→4-GlcN(Glc)**_**2**_**) followed by APTS labeling**. Assays were carried out using CDP (40 μg/ml) at 40 °C with Glc-1-P (disodium salt, 50 mM) and (β-1→4-GlcN(Glc)_2_) (5 mM) in NaOAc buffer (50 mM, pH 5) (all concentrations are final concentrations) for 1 h, followed by heating to 95 °C in a boiling water bath for 5 min and centrifuging at 16,000 g for 5 min. 15 μl of this synthetic reaction were labelled with APTS and analysed by CE. For comparison, CDP oligomerization with (β-1→4-Glc)_3_ (green) is shown: oligomers containing GlcN differ 1 min compared to Glc oligomers, which differ 0.7 min in CE electropherogram. CE was performed under standard conditions. **B. CDP inhibition by (β-1→4-GlcN(Glc)**_**2**_**)**. Assays were carried out using CDP (40 μg/ml) at 40 °C with Glc-1-P (disodium salt, 50 mM) and (β-1→4-Glc)_3_-APTS (0.5 mM) in HEPES buffer (50 mM, pH 7.6) adding (β-1→4-GlcN(Glc)_2_) (5 mM) (all concentrations are final concentrations) for 1.5 h, followed by heating to 95 °C in a boiling water bath for 5 min and centrifuging at 16,000 g for 5 min. CE was performed under standard conditions. (For interpretation of the references to colour in this figure legend, the reader is referred to the web version of this article.)

Kadokawa and co-workers have extensively investigated the flexibility of α-glucan phosphorylases towards the insertion of single or multiple non-cognate monosaccharides into maltooligosaccharides [37], [38], [39], [40], [41], [42]. In particular, α-glucan phosphorylase from potato was able to effect single addition of GlcN onto maltotetraose [38]. Later, a thermostable α-glucan phosphorylase from *Aquifex aeolicus* was shown to randomly incorporate GlcN [37], [41], [42] or GlcA [39], [41] onto maltooligosaccharides to provide new chitin/chitosan/glycosaminoglycan-like glyco-materials, which could find applications in drug delivery. The introduction of a GlcN into cellulose oligomers could confer them with interesting properties, such as pH-responsiveness and improved solubility. Therefore, we tested whether the isolated trisaccharide (β-1→4-GlcN(Glc)_2_), formed by the addition of GlcN onto cellobiose (section 2.3), could subsequently serve as an acceptor for CDP with Glc-1-P as donor. CDP successfully extended the trisaccharide, as shown by MALDI ms: the generated cello-oligomers containing a single GlcN residue (Supplementary Fig. S8, blue) differed by 162 m/z, but had a −1 m/z unit difference compared to pure cello-oligomers (Supplementary Fig. S8, red). To exclude the possibility of the CDP trisaccharide elongation on the reducing Glc, forming a β-1→1 linkage [43], [44], the availability of the reducing end of the generated GlcN-containing cello-oligomers was confirmed APTS-labeling them by reductive amination (Fig. 5A, red). Successful APTS-labeling allowed analysis of the products by CE, where peaks corresponding to cello-oligomers containing GlcN differed by 1 min in retention time in the electropherogram (Fig. 5A, red) compared to peaks of β-1→4-glucan oligomers differing by 0.7 min (Fig. 5A, green). To further confirm that Glc was added at the non-reducing end with β-1→4 linkage rather than onto the reducing end with β-1→1 linkage, reduction of the reducing end with NaBD_4_ was carried out and confirmed by a mass increment of 3 Da observed by MALDI ms (Supplementary Fig. S9). A competition experiment was set up to probe CDP specificity toward GlcN-containing cello-oligosaccharide acceptors. When (β-1→4-GlcN(Glc)_2_) was added in 10 fold excess to (β-1→4-Glc)_3_-APTS, the average extension of (β-1→4-Glc)_3_-APTS reduced substantially, suggesting the ability of GlcN-containing cello-oligosaccharides to compete with β-1→4-glucan oligosaccharides for CDP active site (Fig. 5B).

**Fig. 6:**
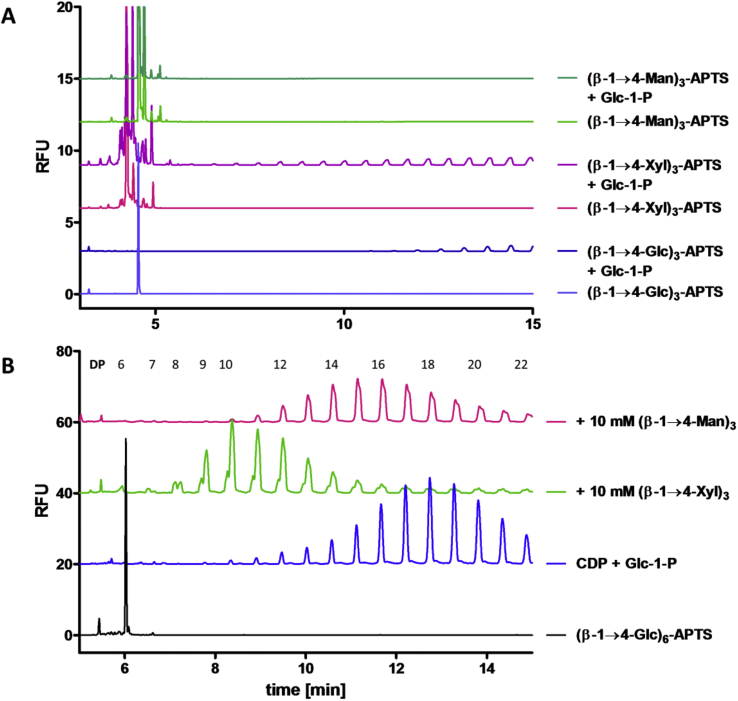
**β-1→4-glycans as CDP acceptors: CE analysis. A. CDP oligomerization of (β-1→4-Xyl)**_**3**_**-APTS and (β-1→4-Man)**_**3**_**-APTS**. Assays were carried out using CDP (5 μg/ml) at 40 °C with Glc-1-P (disodium salt, 10 mM) and APTS-labelled acceptor (13.5 μM) in HEPES buffer (50 mM, pH 7.5) (all concentrations are final concentrations) for 20 min, followed by heating to 95 °C in a boiling water bath for 5 min and centrifuging at 16,000 g for 5 min. CE was performed under standard conditions. **B. CDP inhibition by β-1→4-glycans**. Assays were carried out using CDP (5 μg/ml) at 40 °C with Glc-1-P (disodium salt, 10 mM) and (β-1→4-Glc)_6_-APTS (13.5 μM) in HEPES buffer (50 mM, pH 7.5) adding (β-1→4-Xyl)_3_ (10 mM) and (β-1→4-Man)_3_ (10 mM) (all concentrations are final concentrations), followed by heating to 95 °C in a boiling water bath for 5 min and centrifuging at 16,000 g for 5 min. CE was performed under standard conditions.

Xylotriose (β-1→4-Xyl)_3_ and mannotriose (β-1→4-Man)_3_ were labelled with APTS and assessed as acceptors for CDP (Fig. 6A). (β-1→4-Xyl)_3_-APTS is a reasonable acceptor of Glc forming long oligomers (Fig. 6A, purple), which suggests that the 6-OH is not required in the CDP acceptor binding site, which is consistent with the results from Shintate and co-workers [30]. On the other hand, CDP is not able to transfer glucose from Glc-1-P onto (β-1→4-Man)_3_-APTS and there is no apparent phosphorolysis (Fig. 6A, dark green). This suggests that the acceptor binding site of CDP cannot tolerate an axial 2-OH.

In order to probe CDP specificity toward xylo- and manno-oligosaccharide acceptors, competition experiments were carried out and monitored by CE (Fig. 6B). When a large excess of (β-1→4-Xyl)_3_ was added (10 mM *vs* 13.5 μM APTS-labelled acceptor) (Fig. 6B, green), there was a significant decrease in the average extension of the (β-1→4-Glc)_6_-APTS suggesting binding of xylotriose to the active site of the enzyme. In contrast, addition of (β-1→4-Man)_3_ (10 mM *vs* 13.5 μM APTS-labelled acceptor) had almost no impact on the average extension of the (β-1→4-Glc)_6_-APTS acceptor, indicating that the mannose-containing trisaccharide does not compete for the active site (Fig. 6B, pink).

### 2.6 Apo structure of CDP

**Fig. 7:**
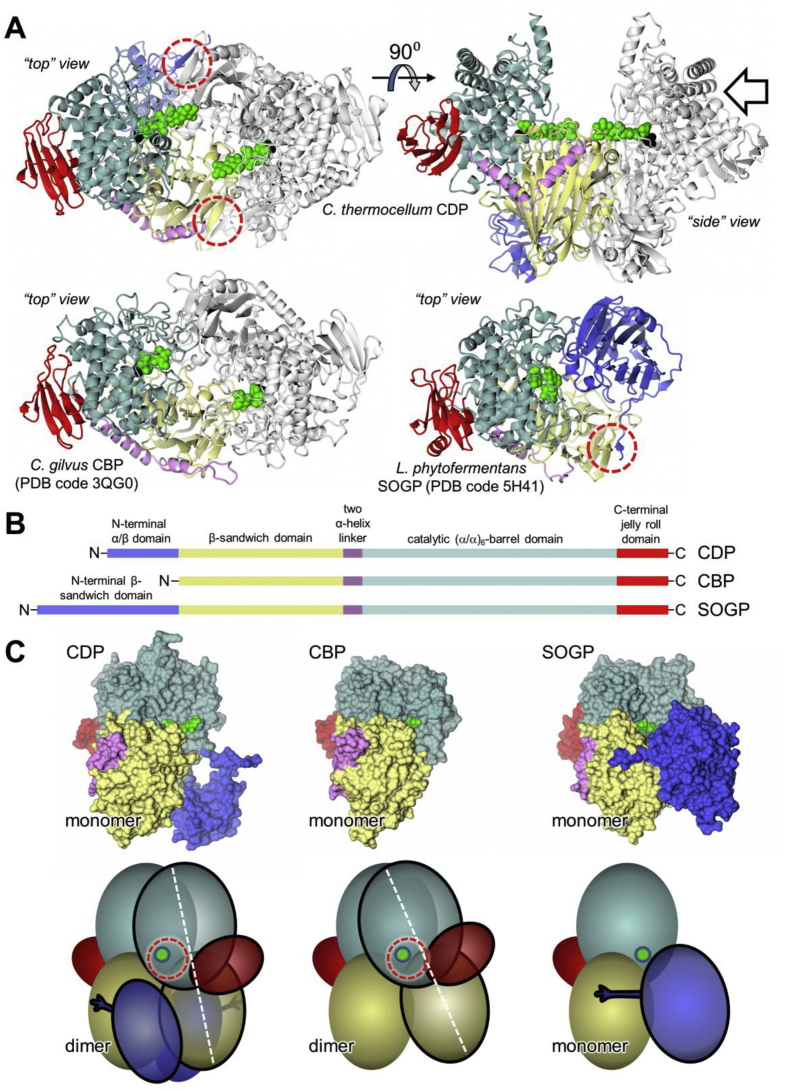
**Comparison of the structures of CDP, CBP and SOGP.** A. The top two images show orthogonal perspectives (“top” and “side” views) of the ligand free structure of *R. thermocellum* CDP homodimer (PDB code 5NZ7) in cartoon representation, this being more complete than the ligand bound structure (PDB code 5NZ8). Superimposed on this are the two copies each of cellotetraose (green spheres) and phosphate (black spheres) taken from the latter (PDB code 5NZ8). One subunit is coloured according to the domains shown schematically in panel B and Supplementary Fig. S10, whilst the other is coloured in grey. The open arrow indicates the direction of view for panel C. The lower two images show corresponding top views for the *C. gilvus* CBP homodimer (PDB code 3QG0) and the *L. phytofermentans* SOGP monomer (PDB code 5H41). The dashed red-circles indicate β-sheet interactions between the N-terminal arms that project from the N-terminal domains of CDP and SOGP and their structurally conserved β-sandwich domains. Note the differences in structure and placement of these N-terminal domains; in SOGP this domain sits in the equivalent place to that occupied by the β-sandwich domains of the opposing subunits in CDP and CBP homodimers and thus explains why this enzyme is monomeric. B. Linear representation of the domain structures of CDP, CBP and SOGP. C. The top panels show molecular surfaces for the monomers of CDP, CBP and SOGP (direction of view shown in part A; looking at the occluded surface in the case of the two that form dimers). The lower panels show simplified representations of the corresponding views of the biological units (which is also a monomer for SOGP), where the various domains are shown as ellipsoids. In the case of the two dimers, the domains of the rear subunit are in solid colour, whilst those in the front subunit are semi-transparent colour and have a black border. The N-terminal extensions in CDP and SOGP are shown as “arms”; for simplicity, the two α-helix linkers are not shown. The principal axis of the front subunit in each of the two dimers is shown as a dotted white line, which emphasises the ∼16° rotation of this subunit in one dimer relative to the other about an axis perpendicular to the page. This has the effect of altering the proximity of the β-sandwich domain of the front subunit to the active site of the rear subunit (green circle) as highlighted by the dashed red-circles. We speculate that by embracing the opposing subunit, the N-terminal arm and domain help to maintain a more open active site in CDP. (For interpretation of the references to colour in this figure legend, the reader is referred to the web version of this article.)

The structure of the CDP was solved to 2.3 Å resolution, revealing two copies of the protein subunit per asymmetric unit forming a clear dimer (Fig. 7A). There are a number of close structural homologues of CDP in the PDB with the top 22 entries giving DALI Z scores in the range 37–40 (and aligning with >73% of the structure) [45], although these represent just five different proteins: cellobiose phosphorylases (CBP) from *Cellulomonas uda* [46], [47], [48], *Cellovibrio gilvus* [27] and *Ruminiclostridium thermocellum* [29], chitobiose phosphorylase from *Vibrio proteolyticus* (ChBP) [49] and cellobionic acid phosphorylase from *Saccharophagus degradans* (CBAP) [50]. Moreover, they all form dimers akin to that of CDP. To simplify the subsequent discussion, given the similarities between CBP, ChBP and CBAP we shall refer to them all as CBP, unless specified otherwise, and a ligand bound structure of *Cellovibrio gilvus* CBP (PDB code 3QG0; contains phosphate, deoxynojirimycin and glucose in the active site) will be the default reference structure (see Supplementary Fig. S10 for a structure-based sequence alignment). Like these enzymes, CDP contains a large β-sandwich domain that forms the majority of the dimer interface, connected by a two α-helix linker to an (α/α)_6_-barrel catalytic domain, and ends with a small peripheral domain that adopts a two layered-jelly roll fold (Fig. 7A and B, refer to Fig. 7B for domain colour-code). Uniquely, CDP has a further ∼120 amino acids at the N-terminus, beginning with an extended arm that leads into a globular domain comprised of a central, five-stranded, mixed β-sheet, flanked by short α-helices; the latter is unrelated to any structurally characterised domain (Fig. 7). Both the α/β domain and the N-terminal arm interact with the β-sandwich domain of the opposing subunit. Indeed, a β-strand (β1) within the arm contributes to the one of the sheets within the β-sandwich (Fig. 7A, dashed red-circle, and C). Together, these additional interactions add substantially to the dimer interfacial area: the total for CDP is ∼4800 Å^2^, as compared to the value of ∼3300 Å^2^ calculated for *Cellovibrio gilvus* CBP (PDB code 3QG0) using the PISA server [51]. Very recently, four structures of β-1→2-oligoglucan phosphorylase (SOGP) from *Lachnoclostridium phytofermentans* were reported [52], which have slightly lower DALI Z scores of ∼32, with only 47% of the structure aligned to CDP and, in contrast to all the aforementioned structures, they are all monomeric. SOGP also differs from these enzymes in that it acts on β-1→2-glucan oligosaccharides rather than β-1→4-glucan oligosaccharides. Like CDP, SOGP also has an extra, albeit much larger (∼250 residues) N-terminal extension, again forming an extended arm and a discrete domain, although the latter resembles the β-sandwich domain common to all these phosphorylases, such that it has two of these domains in tandem (Fig. 7). Remarkably, the additional domain is placed relative to the remainder of the subunit such that it aligns with the β-sandwich domain from the opposing subunit of a superposed CDP/CBP-like dimer, thereby mimicking the dimer interface of these latter enzymes. Moreover, the N-terminal arm of SOGP interacts with the second β-sandwich domain in a similar way to the interaction seen between the N-terminal arm of CDP and the β-sandwich domain of the opposing subunit (Fig. 7A and C).

### 2.7 Ligand bound structure of CDP

**Fig. 8:**
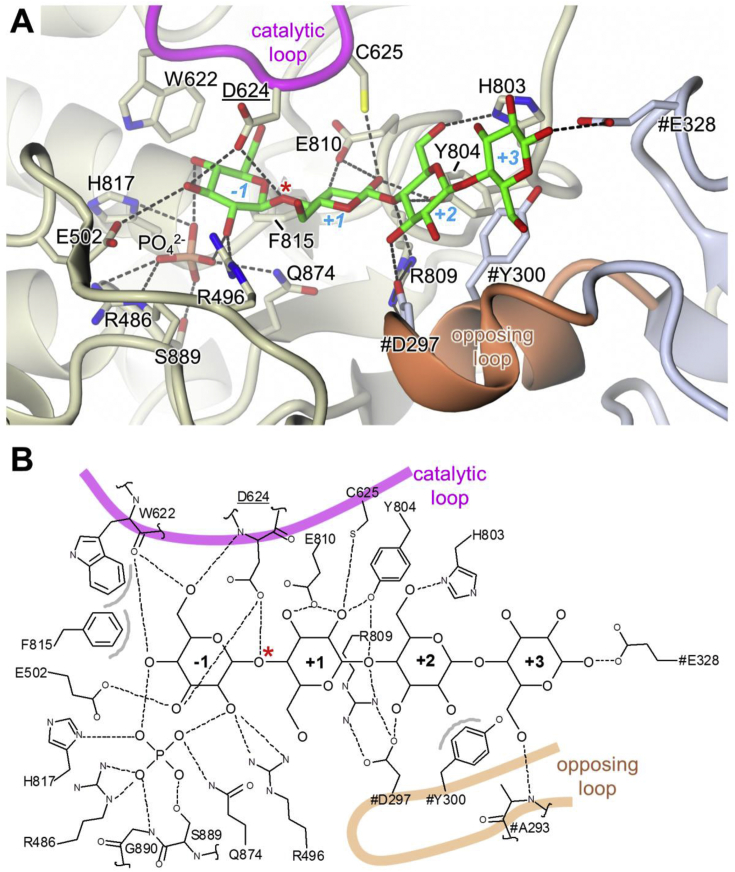
**Details of ligand binding in CDP. A.** Structure of the active site showing the protein backbone in cartoon representation with ligands and neighbouring side-chains as sticks, where ligand carbons are in green, the backbone and side-chain carbons are in cream for the left-hand subunit, and in slate grey for the right-hand subunit. Direct hydrogen bonds with protein side-chains are shown as dashed lines and the catalytic and opposing loops are highlighted. The labels for side-chains from the right-hand subunit are preceded by a hash symbol and the label for the catalytic Asp is underlined. For clarity, some of the foreground detail has been omitted, mainly from the adjacent loop (see Fig. 9A and C), but this does not remove any residues that interact directly with the ligands. The sugar binding subsites are indicated and the scissile glycosidic bond is marked by the red asterisk. The same view is shown in stereo together with omit difference electron density for the bound ligands in Supplementary Fig. S11. **B.** Schematic representation of detail shown in panel A, this time showing all hydrogen bonds including those involving protein backbone atoms. The grey arcs associated with aromatic side-chains indicate van der Waals interactions with the cellotetraose. Hydrogens have been omitted for clarity. (For interpretation of the references to colour in this figure legend, the reader is referred to the web version of this article.)

**Fig. 9:**
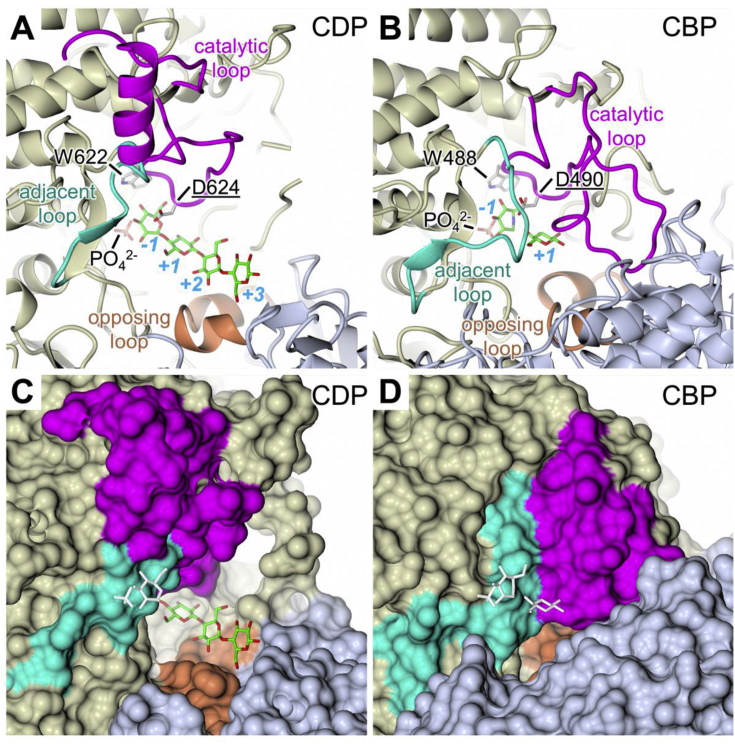
**Comparison of active sites between CDP and CBP**. The active site of CDP is significantly more open than that of CBP due to differences in lengths and conformations of the catalytic and opposing loops, and the relative dispositions of the subunits with the homodimer. The relatively closed dimer interface of CBP drives the opposing loop into the active site of the neighbouring subunit to restrict the length of the acceptor binding site. Panels A and B show cartoon representations with the ligands in stick representation together with the side-chains of the “platform”, Trp and the catalytic Asp. Panels C and D show equivalent views with the protein depicted as a molecular surface. Where ligands are obscured by the protein surface, they are coloured white.

The active site of CDP lies at one end of the (α/α)_6_-barrel of the catalytic domain and the substrate binding site is largely delineated by the loops that connect the outer ring of α-helices to the inner ring. All attempts to co-crystallise CDP with a variety of substrates resulted in poor quality crystals. However, when 10 mM cellohexaose was soaked into a crystal which had been grown in the presence of 10 mM phosphate buffer, a dataset was collected to 3.0 Å resolution revealing additional electron density in the active site cleft, which was interpreted as a cellotetraose molecule with an adjacent phosphate ion. Given the relatively low resolution of this structure we were unable to be certain of the conformation of the sugar rings: we therefore chose to treat them all as β-1→4 linked ^4^C_1_ chairs, which gave a reasonable fit to the electron density with good geometrical parameters. The glucan was oriented such that the non-reducing terminal glycosidic bond was located between the phosphate and Asp624, the expected general acid catalyst (Fig. 8 and Supplementary Fig. S11). Thus, the glucan spanned subsites −1 to +3 of the active site pocket. The donor site is completely buried and does not extend beyond the −1 donor subsite where it is terminated by the side-chain of Trp622, the so-called “hydrophobic platform” residue that is structurally conserved in the close homologues. This is important to exclude water around the region of the scissile bond. The phosphate is located in an adjacent lobe of the active site cleft that is closed off by a sugar bound in the −1 subsite, and thus must be bound before the glucan co-substrate in the phosphorolytic reaction, consistent with it following a sequential Bi Bi mechanism. Beyond subsite +1, the acceptor site opens out and the glucan chain extends across a wide U-shaped canyon formed at the dimer interface (Fig. 9A and C), such that the residues occupying subsites +2 and +3 also interact with side-chains from the β-sandwich domain of the opposing subunit (Fig. 8). Although no electron density is present for sugar residues beyond that occupying the +3 subsite (Supplementary Fig. S11), we cannot rule out the presence of the two further residues expected for cellohexaose. Indeed, this may indicate that the +3 subsite delimits the extent of ordered binding by the acceptor site. There are no large conformational changes between the apo and ligand bound CDP structures (rms deviation of 0.692 Å for a dimer on dimer superposition), although a number of side-chains become reoriented to engage with the substrates. In particular, the carboxylate of the catalytic Asp624 is flipped about the Cα-Cβ bond, to hydrogen bond with O3 of the −1 subsite sugar and the oxygen of the scissile glycosidic linkage; additionally, the adjacent residue within this “catalytic loop”, Cys625, also hydrogen bonds to O2 of the +1 subsite sugar (Fig. 8). Together, these interactions cause the loop, which includes Trp622, to shift towards the bound substrate (Cα-Cα shift of 1.3 Å for Asp624). In general, the majority of the interactions we observe with the −1 and +1 subsite sugars and the phosphate ion are structurally conserved in ligand bound structures of CBP, but the correspondence is weaker for SOGP (*e.g.* in PDB code 5H41), where the architecture necessarily differs because the acceptor is bound in an orientation that is orthogonal to that in the other enzymes [52]. Consistent with the substrate preferences of CBP, its active site is significantly more enclosed than that of CDP (Fig. 9B and D). This is largely due to three structural features. Firstly, the catalytic loops differ in length, being twelve residues longer in CDP (Fig. 9). Whilst the loops are structurally similar up to and including the portion containing Asp624, they adopt completely different conformations after Ile628. In the case of one subunit of apo-CDP, the loop continues away from the active site forming a helix (α17) that projects from the protein surface, before returning to the protein core; in the other subunit, a short section following α17 is disordered. The latter is true for one subunit in ligand-bound CDP (Fig. 9A and C), whereas in the other, substantially more of this loop is disordered, with the exception of the portion bearing Trp622 and Asp624. By contrast in CBP, the catalytic loop is fully ordered, with the C-terminal portion folded over the active site pocket (Fig. 9B and D). Secondly, an “adjacent” loop, which packs against the catalytic loop, also impinges on the active site cleft, but has a lesser impact in CDP as it is five residues shorter than the equivalent loop in CBP. A final significant difference relates to how the acceptor pocket is defined by the opposing subunit, in particular, by two α-helices (α6 and α7) within a loop of the β-sandwich domain. In the more open CDP pocket, two side-chains from this “opposing loop” contribute to the acceptor binding site: Asp297 hydrogen bonds to O3 of the +2 subsite sugar, and Tyr300 forms a stacking interaction with it. There is also a further hydrogen bond to O1 of the +3 subsite sugar, via the side-chain of Glu328 in a different loop of the opposing subunit (Fig. 8 and 9, Supplementary Fig. S11). Compared to CDP, the juxtaposition of the two subunits differs in CBP due to a global rotation about an axis that is perpendicular to, and almost bisects, the two-fold axis relating one half of the dimer to the other (Fig. 7C). In the *Cellovibrio gilvus* CBP (PDB code 3QG0) the rotation is ∼16° (although it is similar for others), and this has the effect of significantly narrowing the canyon between the two subunits and, thereby driving the opposing loop towards the active site (Fig. 9B and D). This foreshortens the acceptor binding pocket, such that in CBP, Gln165 in the equivalent of α7 in the opposing loop, hydrogen bonds to O5 of the +1 subsite and there is no space for further sugar residues beyond this subsite (not shown).

In trying to rationalise these conformational differences, we were drawn to a possible structural role for the unusual N-terminal domain that is unique to CDP. In the homodimer, it is closely associated with the β-sandwich domain of the neighbouring subunit; indeed, together with the N-terminal arm, it appears almost to embrace it. This interaction could have the effect of drawing together the lower portions of the subunits (Fig. 7C), whilst causing the upper portions to move apart, thereby leading to a widening of the canyon that lies adjacent to the active site and allowing larger acceptors to bind.

It is expected that CDP will employ an inverting displacement mechanism (Figs. S12A and B) with the phosphorolytic reaction cleaving a glucose unit from the non-reducing end of a cellodextrin polymer to yield α-D-glucose 1-phosphate. In contrast to the typical glycosyl hydrolase mechanism (Fig. S12C), only a single carboxylate side-chain (Asp624) is required, which acts as the general acid. The reaction proceeds via a nucleophilic attack by the phosphate on the anomeric carbon of the scissile glycosidic bond, and since the phosphate is already ionised at physiological pH, there is no requirement for a general base (*i.e.* a second carboxylate) to initiate the reaction. Concurrently, the general acid donates a proton to the glycosidic oxygen to cleave the bond and release the shortened cellodextrin molecule. Consistent with this mechanism, we note that in the ligand bound structure of CDP, Asp624 is within hydrogen bonding distance of the glycosidic bond oxygen, indicating that it must be protonated. Moreover, the phosphate is appropriately positioned for the nucleophilic attack.

With the benefit of the ligand bound structure of CDP, we can attempt to rationalise substrate preferences. For instance, the 6OH of −1 subsite sugar forms hydrogen bonds with the main-chain carbonyl of Trp622 and the main-chain amide of Asp624, and C6 makes van der Waals contacts with the side chains of both Trp622 and Phe815. Since xylose lacks both C6 and O6, this could explain the poor donor activity with xylose 1-phosphate. Whilst the poor activity with mannose 1-phosphate could result from the switch from an equatorial to an axial configuration for the hydroxyl at C2, with the concomitant loss of hydrogen bonds to the side-chains of Arg496 and Gln874 and to the phosphate, as well as a potential clash of the hydroxyl with the catalytically competent configuration of Asp624. CDP activity with glucosamine 1-phosphate leading mainly to the observed single turnover reaction (see section 2.2, 2.3) may be explained by the stabilisation of the sugar 1-phosphate in the active site due to the ionic interactions between the amino group in GlcN and the −1 subsite and the phosphate group. In addition, the replacement of oxygen with nitrogen at the 2 position of the +1 subsite acceptor sugar (GlcN *vs* Glc) should not alter the binding significantly maintaining all hydrogen bonds with Glu810, Cys625 and Tyr804, as shown by the good acceptor activity of (β-1→4-GlcN(Glc)_2_) toward extension with Glc-1-P (see section 2.5.2).

## 3 Conclusions

Extensive work has previously been carried out on cellobiose phosphorylases to assess the substrate promiscuity and to synthesise disaccharides. Monitoring polymerization reactions is less straightforward, but utilising the combination of resolution and dynamic range provided by capillary electrophoresis and MALDI the long polymers produced by this enzyme could be resolved. Different polymers can be produced using mixed substrates and the relative impact of binding on the turnover of non-natural substrates can give clues to the nature of enzyme-substrate interactions.

CDP can be used to synthesise long β-1→4-glucans which rapidly precipitate out of solution. There is a slow transfer of other sugars, including galactose, mannose, xylose and glucosamine, and competition assays have helped to define the substrate recognition by CDP. While Xyl-1-P and Man-1-P have been shown to be poor CDP donors, GlcN-1-P was shown for the first time to be a relatively good donor for CDP; GlcN-containing oligomers were reasonable CDP acceptors. Moreover, xylotriose was confirmed to be a reasonable acceptor and to outcompete the APTS-labelled β-1→4-glucan, indicating its binding to the active site.

The crystal structure of CDP from *Ruminiclostridium thermocellum* reported herein is the first solved for cellodextrin phosphorylase enzymes. This reveals a novel N-terminal domain that may be involved in adjusting the relative orientations of the two subunits within the homodimer. Together with substantial rearrangements of loops that delineate the active site, this leads to a significantly more open acceptor binding pocket relative to CBP, consistent with the capacity of CDP to accept longer oligomers as acceptor substrates. Together with the substrate binding assays, the crystal structure will help to inform the engineering of CDP for wider use in β-1→4-glucan oligosaccharide synthesis.

## 4 Experimental

### 4.1 General

All synthetic reagents were obtained commercially and used as received, unless otherwise stated. Milli-Q H_2_O was used for the preparation of aqueous buffers. Commercial 2,3,4,6-tetra-*O*-acetyl-α-D-glucopyranosyl fluoride (carbosynth) was deprotected according to standard procedures. All reagents and solvents used for analytical applications were of analytical quality. TLC was performed on precoated slides of Silica Gel 60 F254 (Merck). Reaction products were characterised by Matrix assisted Laser Desorption Ionization-Time of Flight (MALDI-ToF) and electrospray ionization mass spectrometry (ESI-MS), ^1^H, 2D-COSY and 2D-HSQC NMR spectroscopy. Nuclear magnetic resonance spectra were recorded at 298 K on a Bruker Avance III 400 spectrometer, ^1^H spectra at 400 MHz and ^13^C spectra at 101 MHz. Chemical shifts (δ) are reported in parts per million (ppm) with respect to residual HOD signal in D_2_O (δ_H_ 4.79). Coupling constants (*J*) are reported in Hz. NMR signal assignments were made with the aid of COSY and HSQC experiments. MALDI was performed on a Bruker Autoflex Speed using 2,5-dihydroxybenzoic acid (DHB, 10 mg/ml in MeOH + 0.1% TFA) as matrix in positive mode. Enzymatic reactions were desalted prior MALDI sample preparation by addition of mixed bed resin (Sigma) and incubation at rt for 2 min. Samples were typically mixed 1:1 (v/v) with the matrix and spotted on a target plate (Bruker MTP 384 Polished Steel TF Target). Accurate electrospray ionization mass spectra were obtained on a Synapt G2-Si mass spectrometer (Waters, Manchester, UK). Samples were diluted into 50% methanol/0.1% formic acid and infused into the mass spectrometer at 5–10 μl/min using a Harvard Apparatus syringe pump. The mass spectrometer was controlled by Masslynx 4.1 software (Waters). It was operated in resolution and positive ion mode and calibrated using sodium formate. The sample was analysed for 2 min with 1 s MS scan time over the range of 50–1200 m/z (or as appropriate) with 3.5 kV capillary voltage, 40 V cone voltage, 100 °C cone temperature. Leu-enkephalin peptide (1 ng/ml, Waters) was infused at 10 μl/min as a lock mass (m/z 556.2766) and measured every 10 s. Spectra were generated in Masslynx 4.1 by combining a number of scans, and peaks were centred using automatic peak detection with lock mass correction. Ion-exchange chromatography was performed using Bioscale™ Mini Macro-Prep High S cartridge and a step-gradient from 0 to 1 M ammonium bicarbonate buffer (pH 9.4). Compounds were visualised by spraying TLC with orcinol solution (20 mg/ml orcinol monohydrate in EtOH/H_2_SO_4_:H_2_O 75:10:5, v/v), followed by heating. Product containing fractions were combined and reduced to dryness. The residue was co-evaporated repeatedly with methanol to remove residual ammonium bicarbonate. Gel Filtration Chromatography was performed on a Perkin Elmer series 200 equipped with a Toyopearl TSK-HW40S column (90 cm × 1.6 cm), a refractive index detector and a fraction collector. Colourimetric assays were performed in NUNC 96 plates on a BMG labtech FLUOStar Omega microplate reader equipped with suitable absorbance filters.

### 4.2 Expression and purification of CDP

The codon optimised gene for *Ruminiclostridium thermocellum* cellodextrin phosphorylase was synthesised and sub-cloned into the *Bam*HI site of pET15b. An overnight culture of BL21 (DE3) cells containing this plasmid (4 × 1 ml) was used to inoculate 4 × 1 L cultures of LB which were grown at 37 °C until an OD_600_ of ∼0.6. The cultures were then cooled to 30 °C and induced with 1 mM IPTG. After 4 h, the cells were harvested by centrifugation and frozen at −80 °C until required. When required, cell pellets were thawed and re-suspended in 50 ml of lysis buffer (50 mM HEPES, pH 7.5, 100 mM NaCl, 1× Complete™ EDTA-free Protease Inhibitor Cocktail Tablet (Roche), 0.02 mg/ml DNaseI). Cells were lysed using a cell disruptor (one shot mode, 25 kpsi, Constant Systems) and the cell debris removed by centrifugation at 30,000 g (30 min, 4 °C). Protein was purified at 4 °C using an ÄKTAxpress FPLC system (GE Healthcare). The supernatant was passed through a HiTrap Ni-NTA column (5 ml, GE Healthcare), washed with wash buffer (50 mM Tris-HCl, pH 8.0, 0.5 M NaCl, 0.03 M imidazole) and eluted in one step with 50 mM Tris-HCl, pH 8.0, 0.5 M NaCl, 0.5 M imidazole (BioUltra). Further purification was performed by gel filtration using a Superdex S75 26/60 column (GE Healthcare) with GF buffer (50 mM HEPES, pH 7.5, 100 mM NaCl, 3.2 ml/min). Fractions containing CDP were collected (Supplementary Fig. S1), pooled and concentrated to 40 mg/ml using an Amicon Ultra-15 30 kDa MW cut off concentrator. The protein yield was approximately 10 mg/L of culture. The His-tag was not cleaved and the protein was stored in aliquots at −80° until required. Prior to crystallisation, dynamic light scattering was carried out to monitor the solution properties of the purified sample with a DynaPro-Titan molecular-sizing instrument at 20 °C (Wyatt Technology). For *de novo* structure determination, CDP was labelled with selenomethionine (SeMet) by metabolic inhibition [53]. Cells containing plasmid pET15b-CDP were grown in SeMet minimal media (SeMet MM: M9 salts, 0.2% glucose, 2 mM MgSO_4_, 0.1 mM CaCl_2_, 10 mg/l thiamine, 20 ml of amino acid stock) containing 100 μg/ml carbenicillin. The amino acid stock contains Arg, Asp, Glu, Gln, His, Ile, Leu, Lys, Phe, Ser, Thr, Try, Tyr and Val (2 mg/ml). An overnight LB culture of cells (5 ml) was collected by centrifugation (4000 g) and washed with SeMet MM (3 × 5 ml). These cells were then grown in SeMet MM (1 L) at 37 °C until OD600 of 0.6 was reached and then Lys, Phe and Thr (100 mg), Ile, Leu and Val (50 mg) and SeMet (60 mg) were added and the temperature adjusted to 30 °C. After a further 45 min, cells were induced with IPTG (1 mM) and after 16 h the cells were collected by centrifugation. The cells were harvested, stored and purified as described above to give a final yield of 12 mg of purified protein (Supplementary Fig. S2).

### 4.3 APTS labeling of sugars

Sugars were labelled for electrophoresis with 8-aminopyrene-1,3,6-trisulfonic acid (APTS) according to the PACE method [34]. APTS (0.5 mg, 0.2 M in 30% aqueous acetic acid, 5 μl) was mixed with NaBH_3_CN (0.5 mg, 0.8 M in THF, 5 μl). Reducing carbohydrate (1 mg) was dissolved in the mixture and incubated at 37 °C for 18 h or 70 °C for 2 h. The sample was then loaded on to a 30% 38:2 mono:bis acrylamide (Merck) Tris-borate (100 mM, pH 8.2) gel and separated by electrophoresis at 400 V in Tris-borate buffer (100 mM, pH 8.2), water cooled to room temperature. The carbohydrate band (upper) was excised and the labelled sugar was extracted into purified water by grinding using a ceramic bead in a bead mill (Fast Prep FP 120, Thermo) and washing with Milli-Q H_2_O (3 × 30 ml). The borate was removed, either by desalting using a PD-10 column (GE Healthcare) or by evaporating three times from methanol, and the product was quantified using APTS absorption at 455 nm (17,160 1/M/cm) [54].

### 4.4 Capillary electrophoresis with laser induced fluorescence (CE-LIF)

After enzymatic reactions had been carried out using APTS-labelled carbohydrates, samples were placed in boiling water for 5 min and centrifuged for 5 min at 16,000 g, to inactivate and remove proteins. The samples were made up to at least 50 μl and loaded on to an N-CHO coated capillary (50.2 cm, 50 μm) in a PA800 ProteomeLab (Beckman Coulter) by injection at 0.5 psi for 20 s. They were then separated in running buffer (25 mM LiOAc, 0.4% polyethylene oxide, pH 4.75, 20 °C) at 30 kV for 7–45 min and detected using LIF (excitation at 488 nm and detection at 520 nm) [35], [55].

### 4.5 Synthesis of (β-1→4-GlcN(Glc)_2_)

CDP (0.2 mg/ml) was added to a solution of cellobiose (8 mM) and GlcN-1-P (42 mM) in HEPES buffer (50 mM, pH 7.5) (all concentrations are final concentrations). The reaction was incubated at 40 °C for 12 h. After this time, the reaction was quenched in boiling water for 5 min and centrifuged for 1 min at 16,000 g, to inactivate and remove protein. Then, the reaction was diluted with H_2_O and loaded onto a cation exchange cartridge. The product was eluted with 0.05 M ammonium bicarbonate buffer (pH 9.4) and the product containing fractions co-evaporated with methanol to remove the volatile buffer, then freeze-dried. ^1^H NMR (400 MHz; D_2_O) δ: 5.24 (d, *J*_*1′,2′*_ = 3.8 Hz, α−H-1′), 4.68 (d, *J*_*1′,2′*_ = 8.0 Hz, β−H-1′), 4.55 (d, *J*_*1′′,2′′*_ = 8.1 Hz, 1H, H-1′′), 4.49 (d, *J*_*1′′′,2′′′*_ = 8.1 Hz, 1H, H-1′′′), 4.0–3.60 (m, 15H, H-3′, H-3′′, H-3′′′, H-4′, H-4′′, H-4′′′, H-5′, H-5′′, H-5′′′, H-6′, H-6′′, H-6′′′), 3.59 (dd, *J*_*1′,2′*_ = 3.8 Hz, *J*_*2′,3′*_ = 9.87 Hz, α−H-2′), 3.30 (dd, *J*_*1′,2′*_ = 8.0 Hz, *J*_*2′,3′*_ = 8.97 Hz, β−H-2′), 3.37 (H-2′′), 2.72 (H-2′′′). ^13^C NMR (101 MHz; D_2_O) δ: 102.69 (C-1′′′), 102.15 (C-1′′), 95.70 (β−C-1′), 91.75 (α−C-1′), 73.93 (β−C-2′), 72.92 (C-2′′), 56.37 (C-2′′′). TLC: *R*_*f*_ = 0.29 (*i*PA/NH_4_OH/H_2_O 6:3:1); m/z (ESI) 526.1741 [M+Na]^+^, C_18_H_33_NNaO_15_^+^ requires 526.1742.

### 4.6 Acceptor specificity of CDP

The ability for CDP (50 μg/ml) to transfer Glc from Glc-1-P (10 mM) was assayed at 21 °C for 20 min in HEPES (50 mM, pH 7.5). Various acceptors were assayed over a range of concentrations to obtain kinetic parameters (Supplementary Fig. S6). The enzyme activity was measured by phosphate release assay (see section 4.7). The *K*_M_^app^ was calculated for each using GraFit (Erithacus Software Ltd).

### 4.7 Phosphorylase activity assays

All kinetic analyses were performed in the synthetic direction, measuring release of Pi from Glc-1-P. The concentration of released Pi was measured colourimetrically using a method modified from De Groeve et al. [56] Colour solution (75 μl, 0.1 M HCl, 13.6 mM sodium ascorbate) was added to the stopped enzyme reaction (25 μl), containing sodium molybdate, Na_2_MoO_4_ · 2H_2_O (200 mM), in a microtitre plate. After incubating for 5 min at 21 °C, stop solution (75 μl, 68 mM sodium citrate, 2% acetic acid) was added and A_620_ was measured.

### 4.8 Protein crystallisation

A range of commercially available crystallisation screens were set up (Qiagen and Molecular Dimensions) in 96 well MRC plates (Molecular Dimensions). The well solution was dispensed using a Tecan Freedom Evo robot; the drops (0.3 μl protein plus 0.3 μl well solution) were dispensed using an OryxNano robot (Douglas instruments). The protein solution was diluted to 10 mg/ml with GF buffer and filtered through a 0.1 μm filter before use. Plates were placed in a crystal hotel (CrystalPro, TriTek Corp.) at 20 °C and monitored over the course of 4 weeks. Crystallisation hits were optimised in 24-well hanging drop format using 1 ml well solution and drops comprising of 1 μl protein and 1 μl well solution. It was noticed that, when mixing the CDP protein with the crystallisation solution (20% PEG 3350 (w/v), 300 mM KCl, 100 mM HEPES pH 7.5 buffer), a proportion of the protein rapidly precipitated. Subsequently, the protein was pre-precipitated by mixing an equal volume of the crystallisation solution with CDP (10 mg/ml) and incubating on ice for 15 min. After centrifugation at 16,000 g for 1 min, crystallisation drops were set up by mixing 1 μl of the soluble protein with 1 μl of the crystallisation solution. Protein crystals appeared in two days and were transferred to a cryoprotectant solution comprising the crystallisation solution supplemented with 20% (v/v) glycerol using LithoLoops (Molecular Dimensions), before being flash-cooled in liquid nitrogen and stored in Unipuck cassettes (MiTeGen) prior to transport to Diamond Light Source (Oxford, UK).

### 4.9 X-ray data collection and structure solution

Crystals were transferred robotically to the goniostat on beamline I02 at Diamond Light Source (Oxfordshire, UK) and maintained at −173 °C with a Cryojet cryocooler (Oxford Instruments). X-ray diffraction data were recorded using a Pilatus 6M hybrid photon counting detector (Dectris), then integrated using XDS [57], and scaled and merged using AIMLESS [58] via the XIA2 expert system [59]. Crystals belonged to space group P2_1_, with approximate cell parameters of a = 85, b = 152, c = 91 Å and β = 115° and the solvent content was estimated to be 49%, based on two copies of the protein chain (111.6 kDa) in the asymmetric unit (ASU). Experimental phases were determined using PHENIX [60] by combining a native dataset with a SeMet dataset (collected at the Se *K* X-ray absorption edge; wavelength 0.9795 Å), but PHENIX was unable to autobuild the structure from the resultant electron density map. As this point, two copies of a homology model of CDP [based on the structure of *Cellvibrio gilvus* CBP (PDB code 2CQT) [27] and generated by the Phyre2 server (http://www.sbg.bio.ic.ac.uk/phyre2) [61] were manually docked onto a skeletonised version of this map using COOT. After refinement of this preliminary model using REFMAC5 [62], model and experimental phases were combined using SIGMAA [63], then density modified (incorporating two-fold averaging) in PARROT [64], to yield a much improved map at 2.3 Å resolution. This enabled a new model of CDP to be built from scratch using BUCCANEER [65], which was completed by several iterations of manual rebuilding in COOT [66] and restrained refinement in REFMAC5 using isotropic thermal parameters and TLS group definitions obtained from the TLSMD server (http://skuld.bmsc.washington.edu/∼tlsmd/) [67]. Model geometries were validated with MOLPROBITY [68] before submission to the Protein Data Bank. This was used as the starting point for refinement of the complex of CDP with cellohexaose and phosphate against a dataset collected to 3.0 Å resolution. All data collection statistics and model parameters are reported in Table 2.

**Table 2:** X-ray data collection and processing.

Data set	Native	SeMet	Cellohexaose + Pi
Data Collection			
Beamline	I02	I02	I02
Wavelength (Å)	0.9795	0.9795	0.9795
Detector	Pilatus 6M	Pilatus 6M	Pilatus 6M
Resolution range (Å)^a^	50.60–2.30 (2.36–2.30)	83.62–3.50 (3.59–3.50)	27.62–3.00 (3.08–3.00)
Space Group	P2_1_	P2_1_	P2_1_
a, b, c (Å)	84.6, 151.8, 92.0	84.7, 151.8, 92.0	84.8, 153.0, 92.2
α, β, γ (°)	90.0, 114.6, 90.0	90.0, 114.6, 90.0	90.0, 114.4, 90.0
Total observations^a^	522,852 (22,947)	92,835 (6675)	141,679 (10,024)
Unique reflections^a^	93,120 (6720)	26,456 (1937)	41,938 (2981)
Multiplicity^a^	5.6 (3.4)	3.5 (3.4)	3.4 (3.4)
Mean *I*/σ(*I*)^a^	14.3 (2.0)	18.9 (7.1)	12.8 (1.2)
Completeness (%)^a^	99.6 (97.3)	99.0 (99.1)	98.0 (94.8)
*R*_merge_^a^^,^^b^	0.084 (0.759)	0.043 (0.142)	0.064 (1.086)
*R*_meas_^a^^,^^c^	0.102 (1.035)	0.061 (0.197)	0.077 (1.291)
*CC*_½_^a^^,^^d^	0.997 (0.594)	0.995 (0.973)	0.999 (0.425)
Wilson B value (Å^2^)	57.9	72.9	87.8
Refinement			
Reflections: working/free^e^	88,414/4668	–	39,167/2133
*R*_work_^f^	0.191 (0.317)	–	0.210 (0.350)
*R*_free_^f^	0.223 (0.334)	–	0.262 (0.416)
Ramachandran plot: favoured/allowed/disallowed (%)^g^	95.8/3.9/0.3	–	94.1/5.5/0.4
R.m.s. bond deviations (Å)	0.008	–	0.008
R.m.s. angle deviations (°)	1.18	–	1.16
No. of protein residues: ChainA:ChainB	984/984	–	884/974
No. of sugars/phosphates/waters/other	0/0/233/6	–	8/2/0/0
Mean *B*-factors: protein/sugars/phosphates/waters/other/overall (Å^2^)	38.8/−/−/59.6/52.1/39.0	–	114/136/111/−/−/114
PDB accession code	5NZ7	–	5NZ8

a Values for the outer resolution shell are given in parentheses.

b *R*_merge_ = ∑_hkl_ ∑_i_ |I_i_(hkl) − 〈I(hkl)〉|/∑_hkl_ ∑_i_I_i_(hkl).

c *R*_meas_ = ∑_hkl_ [N/(N − 1)]^1/2^ × ∑_i_ |I_i_(hkl) − 〈I(hkl)〉|/∑_hkl_ ∑_i_I_i_(hkl), where I_i_(hkl) is the ith observation of reflection hkl, 〈I(hkl)〉 is the weighted average intensity for all observations i of reflection hkl and N is the number of observations of reflection hkl.

d *CC*_½_ is the correlation coefficient between symmetry-related intensities taken from random halves of the dataset.

e The data set was split into “working” and “free” sets consisting of 95 and 5% of the data, respectively. The free set was not used for refinement.

f The R-factors *R*_work_ and *R*_free_ are calculated as follows: *R* = ∑(| *F*_obs_ - *F*_calc_ |)/∑| *F*_obs_ |, where *F*_obs_ and *F*_calc_ are the observed and calculated structure factor amplitudes, respectively.

g As calculated using MOLPROBITY [68].

All structural figures were prepared using CCP4MG [69], and interfacial areas were determined using the PISA server (http://www.ebi.ac.uk/msd-srv/prot_int/cgi-bin/piserver) [51]. The DYNDOM server (http://fizz.cmp.uea.ac.uk/dyndom/runDyndom.jsp) [70] was used to compare subunit rotations within CDP and CBP dimers.
