# Supplementary material for: Cellodextrin phosphorylase from Ruminiclostridium thermocellum: X-ray crystal structure and substrate specificity analysis
Source: Carbohydr Res. 2017 Nov 8;451:118–32. doi: 10.1016/j.carres.2017.07.005 (PMC5667895; doi:10.1016/j.carres.2017.07.005)
Supplement: Online data [file mmc1.pdf]

Cellodextrin phosphorylase from *Ruminiclostridium thermocellum*: X-ray crystal structure and substrate specificity analysis

Ellis C. O'Neill,[a,b,#] Giulia Pergolizzi,[a,#] Clare E. M. Stevenson,[a] David M. Lawson,[a] Sergey A. Nepogodiev[a], Robert A. Field[a,*]

Table of contents

Table S1. Cellobiose (CBP) and Cellodextrin Phosphorylase (CDP) acceptor specificities.
Table S2. Cellobiose (CBP) and Cellodextrin Phosphorylase (CDP) donor specificities.
Nucleotide sequence of the *E. coli* codon optimised CDP.
Amino acid sequence for *E. coli* codon optimised CDP.
Figure S1. Purification of CDP from *E. coli.*
Figure S2. Purification of SeMet derivatised CDP.
Figure S3. MALDI-ToF and ESI-MS of the isolated (β-1→4-GlcN(Glc)$_2$) product.
Table S3. ^{1}H and ^{13}C NMR data for isolated (β-1→4-GlcN(Glc)$_2$) product in comparison with β-1→4-glucan standards.
Figure S4. 2D-COSY of (β-1→4-GlcN(Glc)$_2$). The spectra were recorded at rt and referenced to HOD (δ_H 4.79).
Figure S5. 2D-HSQC of (β-1→4-GlcN(Glc)$_2$). The spectra were recorded at rt and referenced to HOD (δ_H 4.79).
Figure S6. Acceptor length specificity of CDP.
Figure S7. CDP-catalysed extension of APTS-labelled cello-oligosaccharides.
Figure S8. Comparison of CDP oligomerization for (β-1→4-Glc)$_3$ and (β-1→4-GlcN(Glc)$_2$) with Glc-1-P: MALDI-ToF analysis.
Figure S9. MALDI-ToF analysis of the reduction with $NaBD_4$ of CDP oligomerization products.
Figure S10. Structure-based sequence alignment of *R. thermocellum* CDP (PDB code 5NZ7) with *Cellovibrio gilvus* CBP (PDB code 3QG0).
Figure S11. Details of ligand binding in CDP.
Figure S12. Schematics of (A) phosphorolysis; (B) reverse phosphorolysis with a-D-glucose 1-phosphate; (C) hydrolysis by inverting glycoside hydrolase.

Table S1. Cellobiose (CBP) and Cellodextrin Phosphorylase (CDP) acceptor specificities. Classification based on the non-reducing terminal residue of acceptor.

	Non reducing terminal residue of acceptor		
	**Glucose**	**Other monosaccharides**	**Alditols and alcohols**
**CBP**	D-Glucose[1-6]	L-Fucose[1, 5, 7, 8]	Xylitol[5]
	2-Deoxy-D-glucose[1, 3, 4, 6, 8]	D-Arabinose[1, 5, 7, 8]	Methanol (*etc*…)[9]
	2-Deoxy-2-fluoro-D-glucose[4]	D-Xylose[1-5, 8, 10]	
	3-Deoxy-D-glucose[4, 7]	1,5-anydroglucitol[2, 3, 6]	
	3-Fluoro-3-deoxy-D-glucose[7]	D-Glucuronamide[3]	
	6-Deoxy-D-glucose[2-4, 8]	D-Fructose[5]	
	6-Deoxy-6-fluoro-D-glucose[4]	D-Mannose[1-6, 8]	
	6-*O*-β-D-Glucopyranosyl-D-glucose (gentiobiose)[2, 5, 6, 11]	D-Glucosamine[1-4, 6, 8]	
	6-*O*-α-D-Glucopyranosyl-D-glucose (isomaltose)[5, 6, 11]	D-Altrose[7]	
		L-Galactose[8]	
		D-Allose[7]	
		6-*O*-α-D-Galactopyranosyl-D-glucose (melibiose)[5, 6, 11]	
**CDP**	D-Glucose[12]	PNP β-xyloside[12]	Cellobiitol[13]
	Methyl-β-glucoside[12]	4-*O*-β-D-Xylopyranosyl-D-xylose (xylobiose)[14]	
	Hexyl-β-glucoside[12]	4-*O*-β-D-Xylopyranosyl-D-glucose[14]	
	Octyl-β-glucoside[12]		
	PNP-β-glucoside[12]		
	PNP-β-cellobioside[12]		
	4-*O*-β-D-Glucopyranosyl-D-glucose (cellobiose)[5, 12-17]		
	2-*O*-β-D-Glucopyranosyl-α-D-glucose (sophorose)[5, 16]		
	4-*O*-β-D-Glucopyranosyl-D-altrose[13]		
	4-*O*-β-D-Glucopyranosyl-2-deoxy-D-glucose[13]		
	4-*O*-β-D-Glucopyranosyl-D-mannose[13]		
	4-*O*-β-D-Glucopyranosyl-D-xylose[13, 14]		
	4-*O*-β-D-Glucopyranosyl-1-deoxynojirimytin[18]		
	6-*O*-β-Cellobiosyl-1-deoxynojirimycin[18]		
	3-*O*-β-D-Glucopyranosyl-D-glucose (laminaribiose)[5, 14]		
	4-*O*-β-D-(Glucopyranosyl)$_n$-D-glucose (cellodextrin)[13-15]		

Table S2. Cellobiose (CBP) and Cellodextrin Phosphorylase (CDP) donor specificities.

	**Donor**
**CBP**	α-D-Glc-1-F[4, 5]
	α-D-Glc-1-P[1-8, 11, 19-24]
**CDP**	α-D-Glc-1-F[5]
	α-D-Glc-1-P[5, 12-18, 25-28]
	α-D-Xyl-1-P[14]
	α-D-Gal-1-P[16]

Nucleotide sequence of the *E. coli* codon optimised CDP:

The *cdp* gene from *Ruminiclostridium thermocellum* YM4 strain (GenBank accession number BAB71818) was codon optimised for expression in *E. coli* and synthesised and cloned into pET15b to form pET15b-CDP (BamHI sites underlined).

GGATCCGATGATTACCAAAGTGACGGCACGCAACAATAAAATTACCCCGGTGGAACTGCTGAACCAAAAATTCGGCAATAA
AATCAACCTGGGCAACTTTGCAGACGCTGTGTTCACCGATGCGGCCTTTAAAAATGTTGCGGGCATTGCCAACCTGCCGATG
AAAGCGCCGGTGATGCAGGTTCTGATGGAAAATTGCATCGTTTCGAAATATCTGAAACAATTTGTCCCGGATCGTAGCGTCT
GTTTCGTGGAAGAAGGCCAGAAATTTTACATTGTGCTGGAAGACGGTCAAAAAATCGAAGTGCCGGAAGATGTTAACAAAG
CACTGAAAGCTACCGTTTCAGATGTCAAACATTGGGCAGGCTATCTGACGGAAGACGGTGAACACGTTATTGATCTGCTGAA
ACCGGCTCCGGGTCCGCATTTCTACGTCAATCTGCTGATCGGCAACCGTCTGGGTTTTAAACGCACCCTGCAGACCACGCCGA
AATCTGTGGTTGATCGTTTCGGCCGCGGTTCTTTTCGTAGTCACGCAGCTACCCAGGTTCTGGCAACGCGTTTTGACATGCGC
CAAGAAGAAAATGGCTTCCCGGCTAACCGCCAGTTTTATCTGTACGAAGATGGTAAACAAATCTTTTATAGCGCACTGATTG
ATGACAATATCGTGGAAGCTACCTGCAAACACTCTTGTAACCGTACCGTTATCAAATACAAAACGGCATGCAATCTGGAAAT
TACCCGCACGATCTTCCTGGTGCCGCACAAAAAAGGCTTTCCGCTGGCGACCGAACTGCAGCGTATTGAAATCAAAAATGCG
TCCGATAAAGCCCGCAACCTGTCAATTACCTATACGGGCATGTTCGGCACGGGTGCGGTTCATGCCATTTTTGAAGACGTGAC
CTATACGAATGTTATCATGCAGTCTGCGGCCCTGTACAACGATAAAGGCGAATTTATTGGTATCACCCCGGACTATTACCCGG
AAGAATTTAAACAGGATACCCGTTTTGTCACGATGATTGTGCGCAACGGCGATGAAAAATCCTTTCCGCAATCATTCTGCACC
GATTATAATGACTTTGTGGGCACCGGTACGCTGGAACACCCGGCGGGCGGTTGTAATCTGAACAATAAACTGAACCGTAAAG
GTCCGGGCTTTTTCGCACTGGGTGCTCCGTTCACCGTCGAACCGGGTAAAACGGTGATTATCGATACCTTTACGGGCCTGAGC
TCTAGTAAAGACAACGAAAATTATAGCGATGCCGTGATGCTGCGTGAACTGGATAATCTGCTGCGCTACTTCGAAAAATCGG
AAAGCGTTGAAGAAACCCTGAACGAAATCATCAACTTCCATGAAAACTACGGTAAATACTTCCAGTTCAACACGGGCAATAA
ACTGTTTGATAGTGGTTTCAACCGTAATCTGGCATTCCAGGTGCTGTATCAAACCTTCATGTCTCGCAGTTTTGGCCAGACGC
AAAAAGGTTACCGTGAAATTGGCTTTCGCGAAATCCAGGACCTGTTCGCCTCCATGTACTACTTCATCAACATCGGCTATCAA
GATTTCGTTAAAGAACTGCTGTTTGAATGGACCGCAAATGTCTACAAAATGGGTTACGCTAACCATAACTTCTATTGGGTGGG
CAAACAGCCGGGTCTGTACTCCGATGACTCACTGTGGCTGCTGCAAGCGTATTACCGCTATATTATCTACACCAAAGACACG
AGCGTTCTGAACGAAGAAGTCCCGGTGGCGGATGGCAACAATGAAAAACGTGCCGTCCGCGAAACCCTGAAAGCAATTATC
CAGTATTCGGCTTGCATTAGCGTGGGTGACCACGGTCTGCCGCTGCTGGATCTGGCCGACTGGAATGATTGTCTGAAAATTGC
GTCCAACTCAATCGATGGTGCCACCAAAGAAAAACTGTATTACGAACAGCTGAAGAAAACCAACGGCAAATATGGTGACCG
TTTCATGAGCGATTACTCGGAAAGCGTGATGAATGCGTTTCTGCTGAAACTGGCCATTGATCATCTGGCGGAAATCGCCACCC
TGGATAATGACACGCAGCTGGCGCAGCAAATGTCGGAACTGAGCAAAGAAGTTACCGATCGCATTCAAAAACACGCATGGA
AAGAAAACTTTTTCGCTCGTGTCCTGATCAACCGCTATAAAGACGGCTCTTATACCTACCTGGGCGCGAAAGGTGATAAACT
GAGTGCCGACCCGAATATTGATGGTGTGTATTTTCTGAACAGTTTCGCGTGGTCTGTTCTGAGTGACGTCGCAACCGATGAAC
AGATTGCTATCATGGTTGATGTCATCAAAAAACATCTGCTGACCCCGTATGGTCTGCGTCTGGTTACGCCGGCCGACCTGAAT
AAAATCGCAAACGATACCGCTACGGGTCATTACTTTTTCGGCGATCGCGAAAACGGTGCAGTCTTTAAACACGCGTCCATGA
TGGCCGTGGCAGCTCTGATTAAAGCGGCCAAAAAAGTGAAAGACAATGAACTGGCGAAAGAAATGGCACGTATTGCTTATT
TCATGATCGATCTGGTCCTGCCGTACAAAAACCTGGAAAATCCGTTTCAGGTGGCCGGCAACCCGCGCATTTGCACCCAATA
TATCAATACCGATACGGGCGAAAACATTGGTCCGCTGCTGTCCGGCACCGCAACGTGGCTGAACCTGAATCTGATTTCACTG
GCCGGCATCGAATATACCCGTGATGGTATTTCGTTCAACCCGATCCTGCGCGAAGAAGAAACCCAGCTGAATTTTACGCTGA
AAGCGCCGAAATGTTCTTACAAATTCAGTATCACCAAACCGGTGGGCTTTGCCCGCATGGAATCCTCAGAATACGAACTGTT
TGTTGACGGTCAGAAAATCGATAATACCGTTATTCCGATGTACACCGACGAAAAAGAACATATTGTCACCCTGAAATTCAAA
TGAGGATCC

Amino acid sequence encoded in the CDP open reading frame of pET15b-CDP
(Purification tag highlighted in blue).

MGSSHHHHHHSSGLVPRGSHMLEDPMITKVTARNNKITPVELLNQKFGNKINLGNFADAVFTDAAFKNVAGIANLPMKAPVMQV
LMENCIVSKYLKQFVPDRSVCFVEEGQKFYIVLEDGQKIEVPEDVNKALKATVSDVKHWAGYLTEDGEHVIDLLKPAPGPHFYVN
LLIGNRLGFKRTLQTTPKSVVDRFGRGSFRSHAATQVLATRFDMRQEENGFPANRQFYLYEDGKQIFYSALIDDNIVEATCKHSCN
RTVIKYKTACNLEITRTIFLVPHKKGFPLATELQRIEIKNASDKARNLSITYTGMFGTGAVHAIFEDVTYTNVIMQSAALYNDKGEFI
GITPDYYPEEFKQDTRFVTMIVRNGDEKSFPQSFCTDYNDFVGTGTLEHPAGGCNLNNKLNRKGPGFFALGAPFTVEPGKTVIIDTF
TGLSSSKDNENYSDAVMLRELDNLLRYFEKSESVEETLNEIINFHENYGKYFQFNTGNKLFDSGFNRNLAFQVLYQTFMSRSFGQT
QKGYREIGFREIQDLFASMYYFINIGYQDFVKELLFEWTANVYKMGYANHNFYWVGKQPGLYSDDSLWLLQAYYRYIIYTKDTS
VLNEEVPVADGNNEKRAVRETLKAIIQYSACISVGDHGLPLLDLADWNDCLKIASNSIDGATKEKLYYEQLKKTNGKYGDRFMSD
YSESVMNAFLLKLAIDHLAEIATLDNDTQLAQQMSELSKEVTDRIQKHAWKENFFARVLINRYKDGSYTYLGAKGDKLSADPNID
GVYFLNSFAWSVLSDVATDEQIAIMVDVIKKHLLTPYGLRLVTPADLNKIANDTATGHYFFGDRENGAVFKHASMMAVAALIKA
AKKVKDNELAKEMARIAYFMIDLVLPYKNLENPFQVAGNPRICTQYINTDTGENIGPLLSGTATWLNLNLISLAGIEYTRDGISFNPI
LREEETQLNFTLKAPKCSYKFSITKPVGFARMESSEYELFVDGQKIDNTVIPMYTDEKEHIVTLKFK*

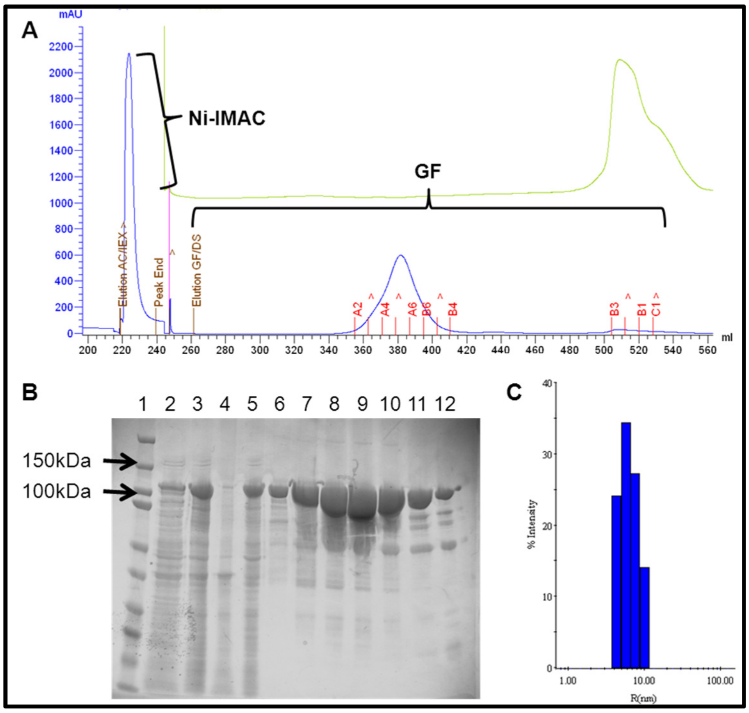

Figure S1. Purification of CDP from *E. coli.* A. Purification of CDP by the standard protein purification protocol (see Experimental 5.2). CDP was eluted from the Ni-IMAC by single step increase of imidazole at 220 ml. The whole peak was injected on the gel filtration column at 260ml to give a single species (A2-B4) at 120 ml elution volume, as judged by A_{280} (blue), with some low molecular weight contaminants eluting in the column volume, as indicated by the conductivity trace (green). **B. SDS-PAGE of the fractions from GF shows a major band, consistent with the calculated MW of 114 kDa.** Lane 1: Kaleidoscope protein standards (Bio-Rad). Lane 2: total cell extract of uninduced cells. Lane 3: total cell extract of induced cells. Lane 4: insoluble induced cell material. Lane 5: soluble induced cell lysate. Lane 6-12: fractions A2-B5 from GF. **C. Dynamic light scattering analysis of CDP.** DLS analysis of CDP (0.1 mg/ml) shows the protein is present as a monodisperse species with a radius of 6.6 nm, which corresponds to a molecular weight of ≈ 277 KDa, comparable to the molecular weight of CDP dimer.

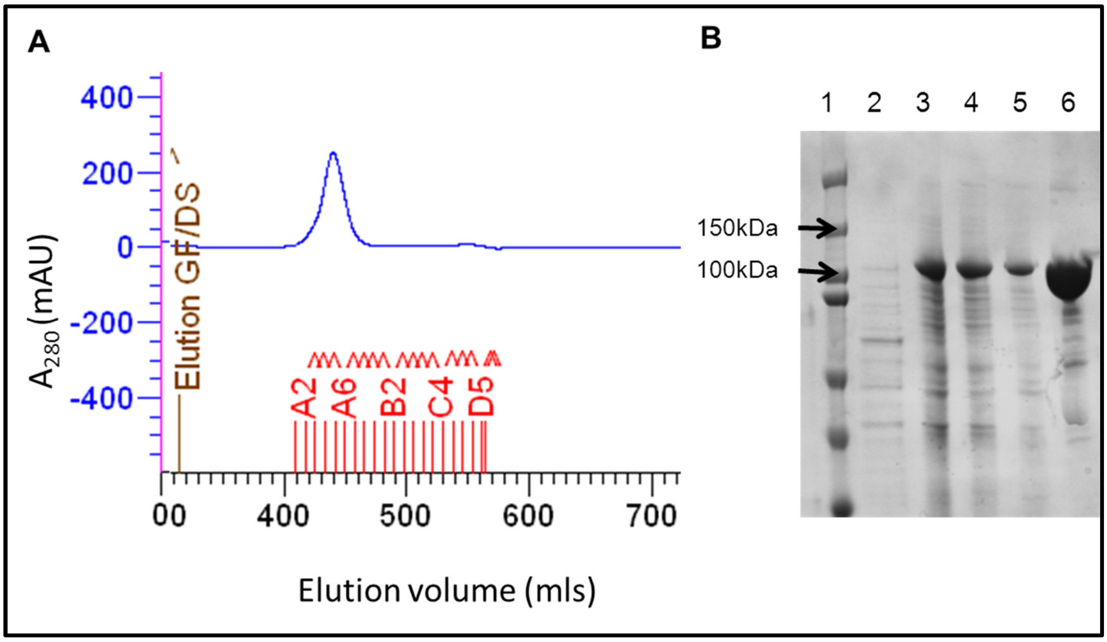

Figure S2. Purification of SeMet derivatised CDP. **A**. GF purification of SeMet-CDP after Ni-IMAC shows a single species, eluting at 120 ml. **B**. **SDS-PAGE of protein purification.** Lane 1: Kaleidoscope protein standards (Bio-Rad). Lane 2: total cell extract of uninduced cells: Lane 3: total cell extract of induced cells. Lane 4: insoluble cell material. Lane 5: soluble cell lysate. Lane 6: purified protein, showing a major band running just over 100 kDa.

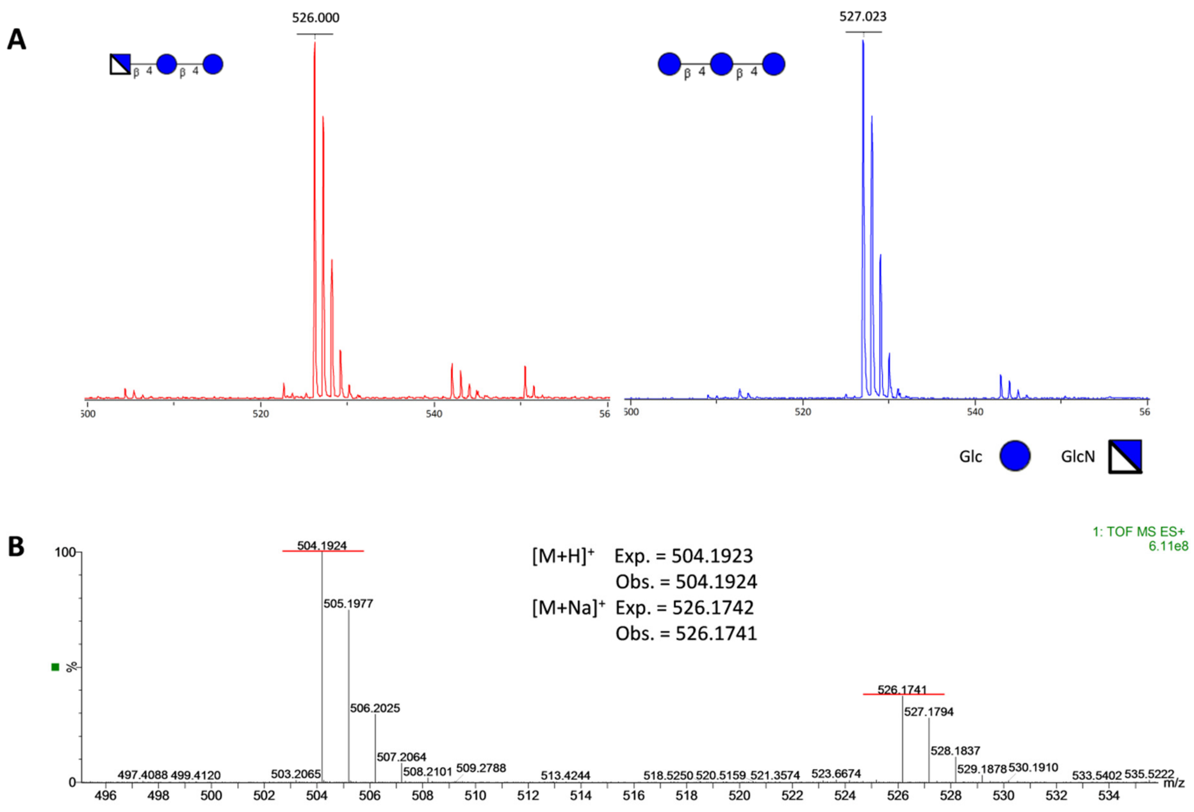

Figure S3. MALDI-ToF and ESI-MS of the isolated (β-1→4-GlcN(Glc)$_2$) product. A. MALDI-ToF MS of (β-1→4-GlcN(Glc)$_2$) (left, red) and (β-1→4-Glc)$_3$ standard (right, blue). Operated in positive ion mode with DHB as matrix. **B. ESI-MS of (β-1→4-GlcN(Glc)$_2$).** Operated in resolution and positive ion mode, and calibrated with sodium formate.

Table S3. ^{1}H and ^{13}C NMR data for isolated (β-1→4-GlcN(Glc)$_2$) product in comparison with β-1→4-glucan standards. The spectra were recorded at rt and referenced to HOD (δ_H 4.79).

	H-1′		H-1″		H-1‴		H-2′	H-2″	H-2‴
	ppm	$J_{1,2}$ [Hz]	ppm	$J_{1',2'}$ [Hz]	ppm	$J_{1'',2''}$ [Hz]	ppm	ppm	ppm
**(β-1→4-Glc)$_3$**	5.23 (α) d<br>4.67 (β) d	3.6<br>8.0	4.56 (β) d	8.1	4.52 (β) d	8.1	3.59 (α)<br>3.30 (β)	3.37	3.33
**(β-1→4-GlcN(Glc)$_2$)**	5.24 (α) d<br>4.68 (β) d	3.8<br>8.0	4.55 (β)	8.1	4.49 (β)	8.1	3.59 (α)<br>3.30 (β)	3.37	2.72
**(β-1→4-Glc)$_2$**	5.24 (α) d<br>4.68 (β) d	3.7<br>8.0	4.53 (β) d	7.9	-	-	3.59 (α)<br>3.30 (β)	3.33	-

	C-1′	C-1″	C-1‴	C-2′	C-2″	C-2‴
**(β-1→4-Glc)$_3$**	91.80 (α)<br>95.73 (β)	102.32	102.54	71.21 (α)<br>73.88 (β)	72.93	73.12
**(β-1→4-GlcN(Glc)$_2$)**	91.75 (α)<br>95.70 (β)	102.15	102.69	73.93 (β)	72.92	56.37
**(β-1→4-Glc)$_2$**	91.80 (α)<br>95.73 (β)	102.54<br>102.51	-	71.20 (α)<br>73.87 (β)	73.15	-

The symbols ′, ″ and ‴ denote the first, second and third glycosyl residue from the reducing end, respectively.

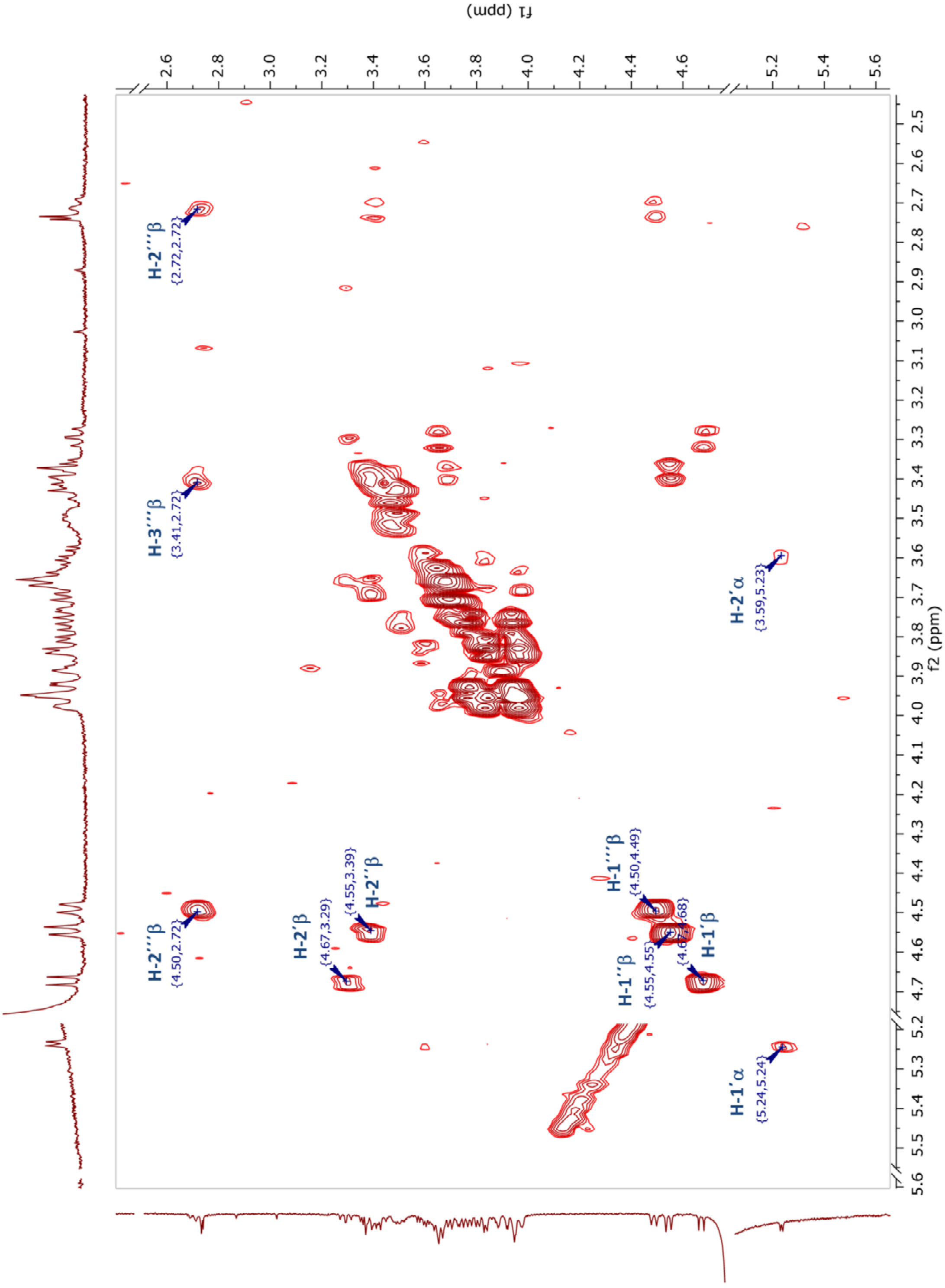

Figure S4. 2D-COSY of (β-1→4-GlcN(Glc)$_2$). The spectra were recorded at rt and referenced to HOD (δ_H 4.79). HOD signal is omitted for clarity.

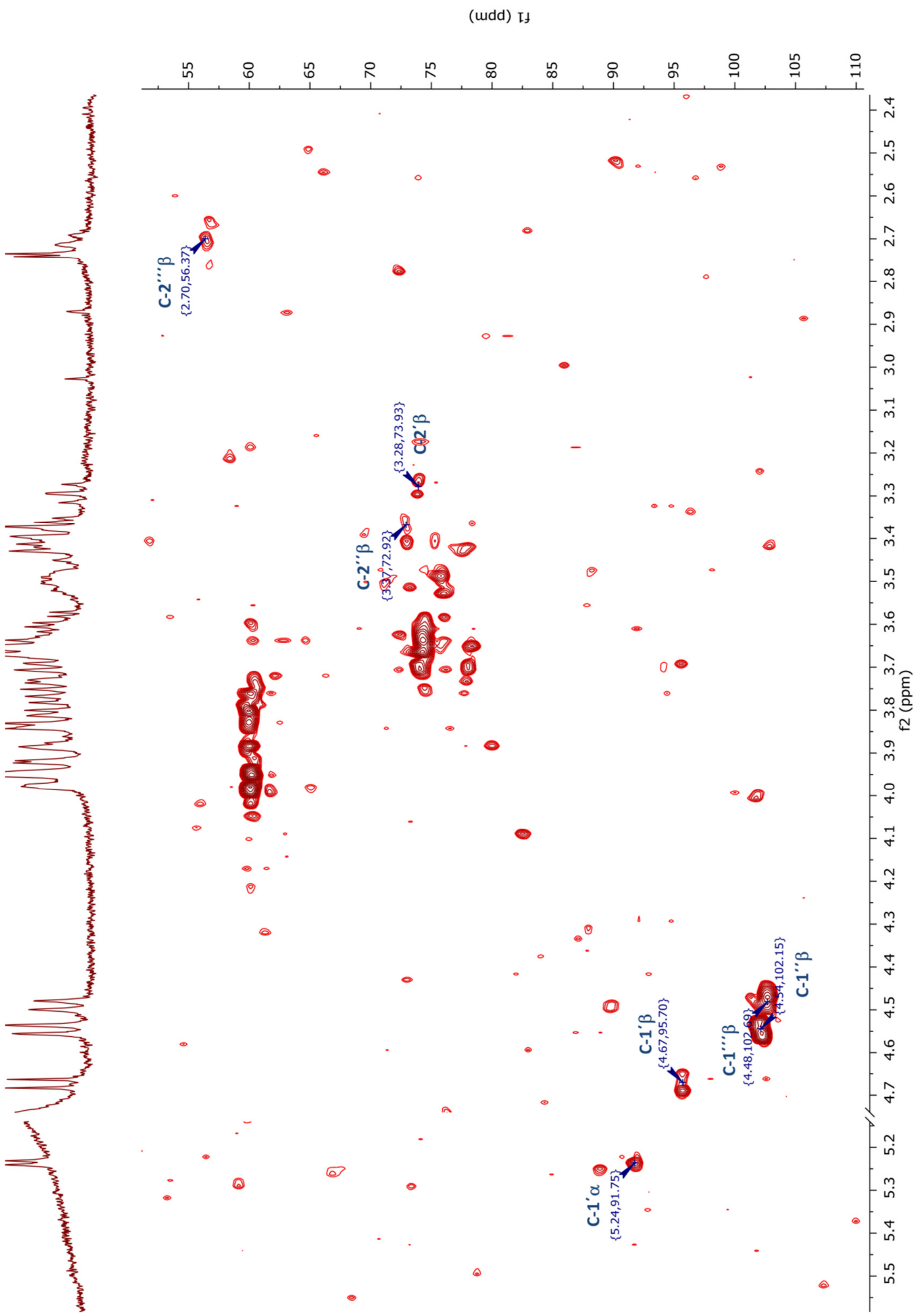

Figure S5. 2D-HSQC of (β-1→4-GlcN(Glc)$_2$). The spectra were recorded at rt and referenced to HOD (δ_H 4.79). HOD signal is omitted for clarity.

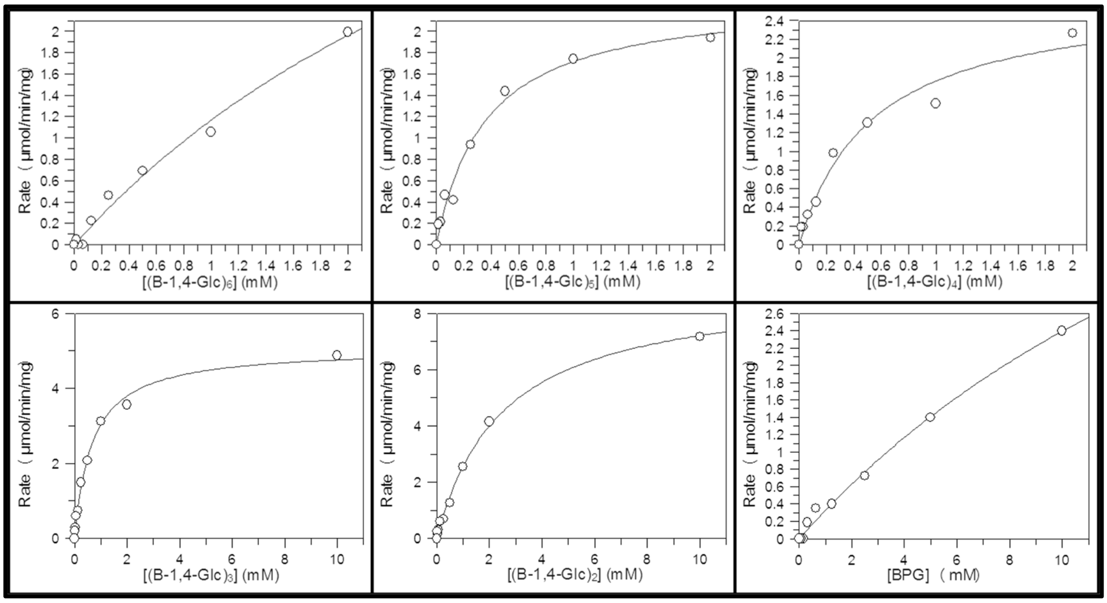

Figure S6. Acceptor length specificity of CDP. The enzyme activity was measured by phosphate release assay over a range of acceptor concentrations. The K_M^{app} was calculated for each acceptor using GraFit (Erithacus Software Ltd).

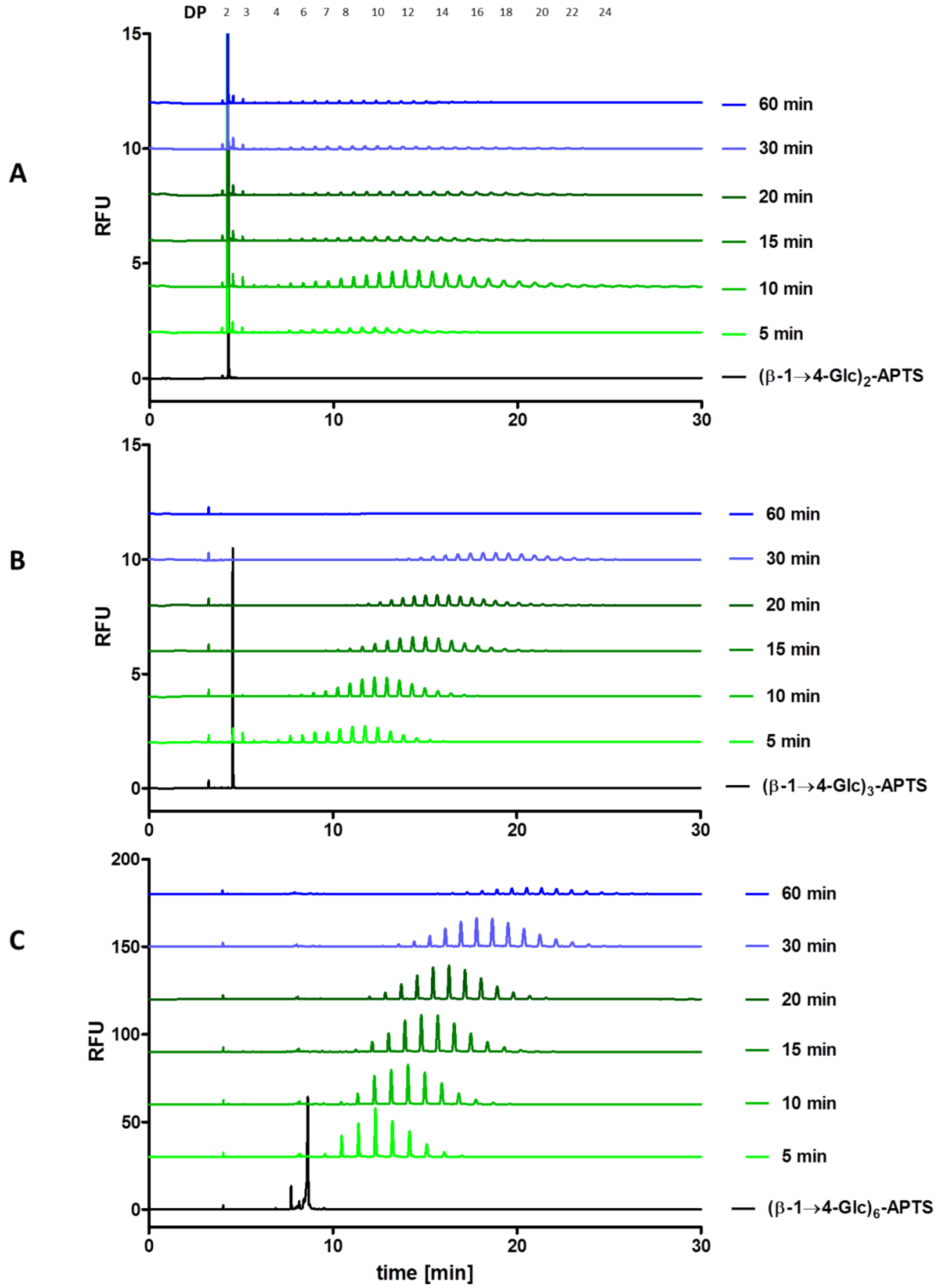

Figure S7. CDP catalyses extension of APTS-labelled cello-oligosaccharides. A. Extension of (β-1→4-Glc)$_2$-APTS. B. Extension of (β-1→4-Glc)$_3$-APTS. C. Extension of (β-1→4-Glc)$_6$-APTS. Assays were carried out using CDP (5 µg/ml) at 40 °C with Glc-1-P (disodium salt, 10 mM) and APTS-labelled acceptor (2 µM) in HEPES buffer (50 mM, pH 7.5) followed by heating to 95 °C in a boiling water bath for 5 min and centrifuging at 16,000 g for 5 min. CE was performed under standard conditions.

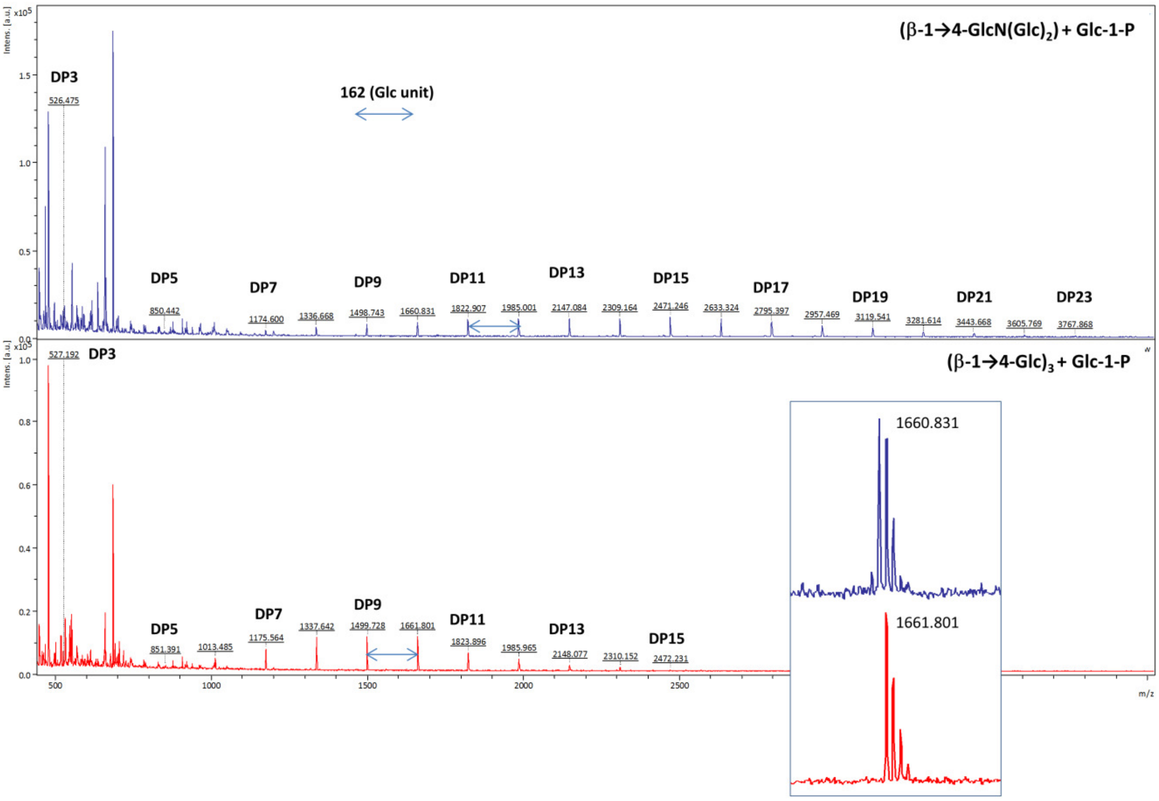

Figure S8. Comparison of CDP oligomerization for (β-1→4-Glc)$_3$ and (β-1→4-GlcN(Glc)$_2$) with Glc-1-P: MALDI-ToF analysis. Assays were carried out using CDP (75 µg/ml) at 40 °C with Glc-1-P (disodium salt, 40 mM) and acceptors (2.5 mM) in NaOAc buffer (50 mM, pH 5) (all concentrations are final concentrations), followed by heating to 95 °C in a boiling water bath for 5 min and centrifuging at 16,000 g for 5 min. The samples were treated with mixed bed resin for salts removal before MALDI analysis. DHB matrix used, positive mode.

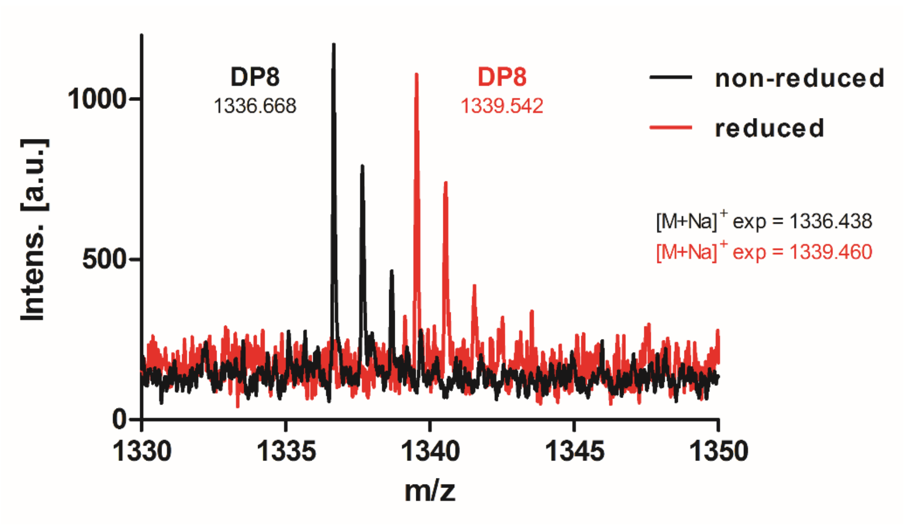

Figure S9. MALDI-ToF analysis of the reduction with NaBD$_4$ of CDP oligomerization products. The mass spectra show the mass of DP8 product with (red) or without (black) reduction with NaBD$_4$. A mass increment of 3 Da is diagnostic for deuterium addition at the reducing end of the oligosaccharide. Spectra of products of DP5 to 10 were similarly recorded.

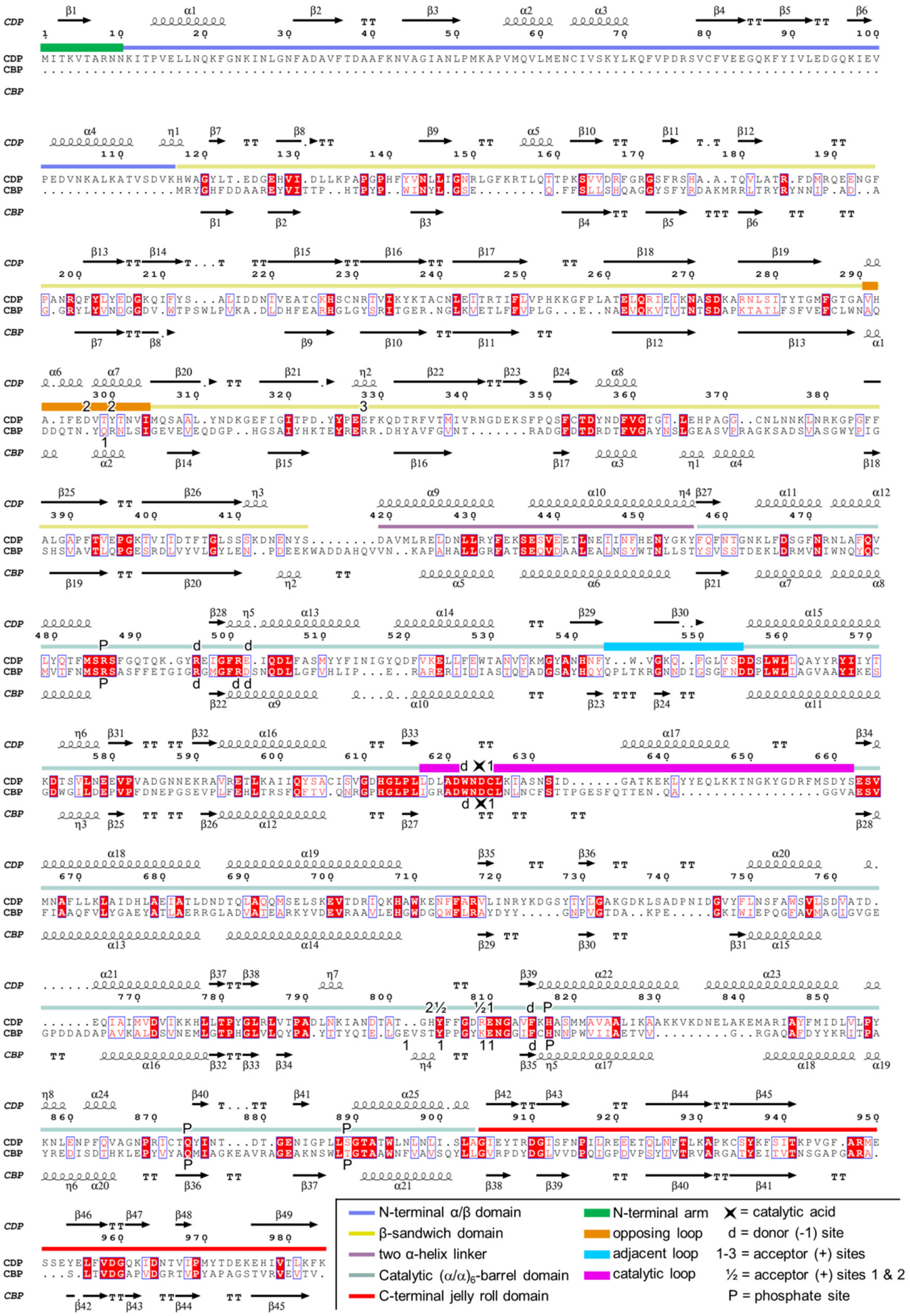

Figure S10. Structure-based sequence alignment of *R. thermocellum* CDP (PDB code 5NZ7) with *Cellovibrio gilvus* CBP (PDB code 3QG0). Shown are the full native sequences, which were aligned using the PDBeFold server (http://www.ebi.ac.uk/msd-srv/ssm/)[29] then displayed using ESPript3.0 (http://espript.ibcp.fr/ESPript/ESPript/)[30]. Strictly conserved residues are highlighted with red shaded boxes, and semi-conserved residues are coloured red. Secondary structure elements for CDP are shown above the alignment, with those for CBP below, where α = α helix, β = β strand, η = 3_{10} helix, TT = β turn. The extents of the various domains in CDP are indicated by the colour-coded thin horizontal bars; the thicker bars highlight specific regions that are referred to in the text. The annotations above and below the sequences indicate amino acid side-chains implicated in catalysis or binding substrates, and their specific roles.

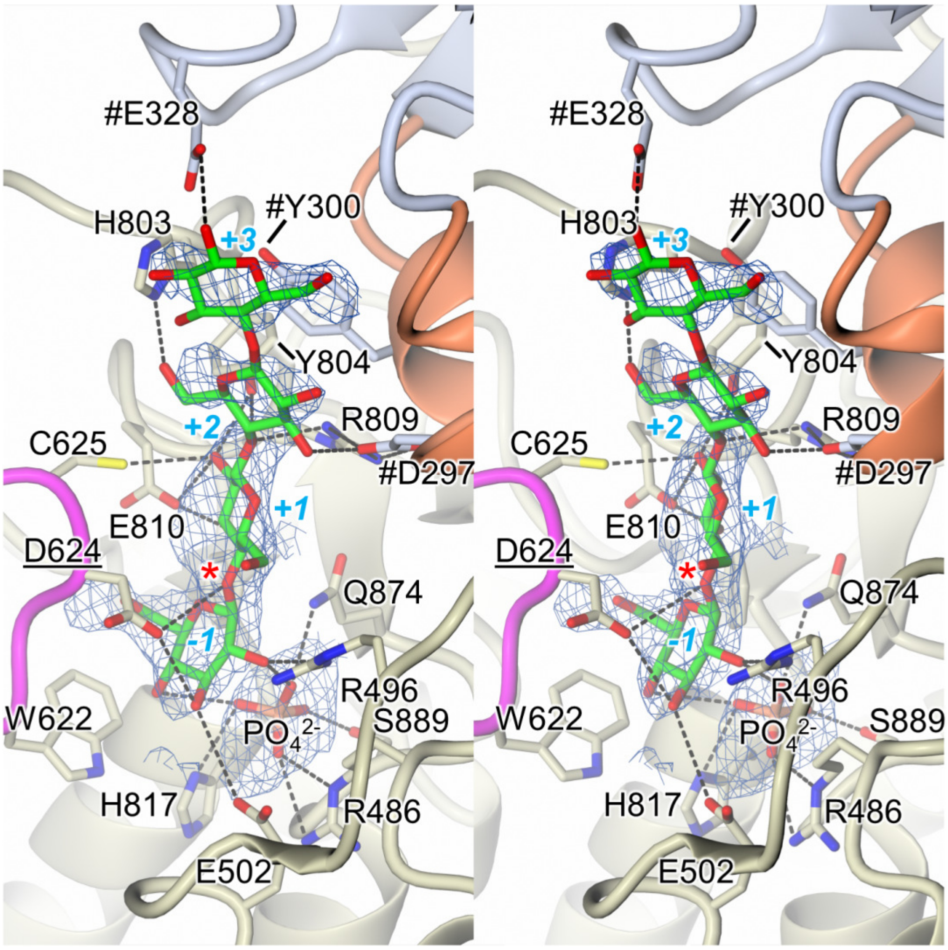

Figure S11. Details of ligand binding in CDP. Stereoview of the active site showing the protein backbone in cartoon representation with ligands and neighbouring side-chains as sticks, where ligand carbons are in green, the backbone and side-chain carbons are in cream for the lower subunit, and in slate grey for the upper subunit. Direct hydrogen bonds with protein side-chains are shown as dashed lines and the catalytic and opposing loops are highlighted. The labels for side-chains from the upper subunit are preceded by a hash symbol and the label for the catalytic Asp is underlined. For clarity, some of the foreground detail has been omitted, mainly from the adjacent loop (see Fig. 9A and C), but this does not remove any residues that interact directly with the ligands. The sugar binding subsites are indicated and the scissile glycosidic bond is marked by the red asterisk. Also shown in blue mesh, is an omit *mFobs-dFcalc* difference electron density map (3.0 Å resolution; contoured at ~3σ) generated for the bound ligands using phases calculated from the final model without these ligands after the application of small random shifts to the atomic coordinates, re-setting temperature factors, and re-refining to convergence.

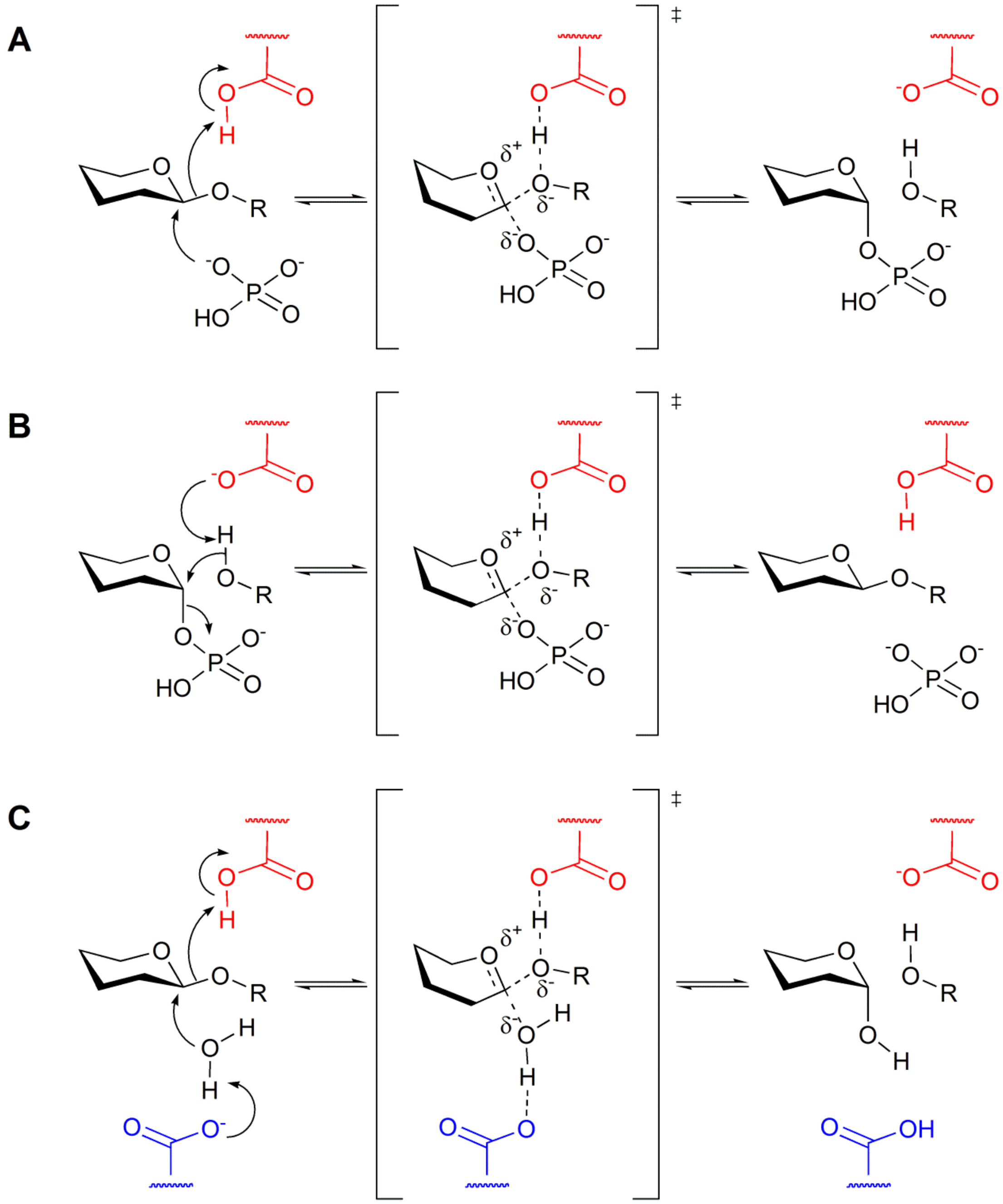

Figure S12. Schematics of (A) phosphorolysis; (B) reverse phosphorolysis with α-D-Glc-1-phosphate; (C) hydrolysis by inverting glycoside hydrolase. Highlighted in red is the aspartate involved in glycoside phosphorylase reaction mechanism. Highlighted in blue is the second aspartate required by glycoside hydrolases.

References:

[1] K. Tanaka, T. Kawaguchi, Y. Imada, T. Ooi, M. Arai, J. Ferment. Bioeng., 79 (1995) 212-216.

[2] K. Hamura, W. Saburi, S. Abe, N. Morimoto, H. Taguchi, H. Mori, H. Matsui, Biosci., Biotechnol., Biochem., 76 (2012) 812-818.

[3] Y.-K. Kim, M. Kitaoka, M. Krishnareddy, Y. Mori, K. Hayashi, J. Biochem., 132 (2002) 197-203.

[4] B. Nidetzky, C. Eis, M. Albert, Biochem. J., 351 (2000) 649-659.

[5] H. Nakai, M. Abou Hachem, B.O. Petersen, Y. Westphal, K. Mannerstedt, M.J. Baumann, A. Dilokpimol, H.A. Schols, J.O. Duus, B. Svensson, Biochimie, 92 (2010) 1818-1826.

[6] M. Hidaka, M. Kitaoka, K. Hayashi, T. Wakagi, H. Shoun, S. Fushinobu, Biochem. J., 398 (2006) 37-43.

[7] A. Percy, H. Ono, D. Watt, K. Hayashi, Carbohydr. Res., 305 (1998) 543-548.

[8] J.K. Alexander, J. Biol. Chem., 243 (1968) 2899-2904.

[9] K. Kino, R. Satake, T. Morimatsu, S. Kuratsu, Y. Shimizu, M. Sato, K. Kirimura, Biosci., Biotechnol., Biochem., 72 (2008) 2415-2417.

[10] M. Kitaoka, H. Taniguchi, T. Sasaki, Appl. Microbiol. Biotechnol., 34 (1990) 178-182.

[11] A. Percy, H. Ono, K. Hayashi, Carbohydr. Res., 308 (1998) 423-429.

[12] G. Hai Tran, T. Desmet, M.R.M. De Groeve, W. Soetaert, Biotechnol. Prog., 27 (2011) 326-332.

[13] K. Sheth, J.K. Alexander, J. Biol. Chem., 244 (1969) 457-464.

[14] K. Shintate, M. Kitaoka, Y.-K. Kim, K. Hayashi, Carbohydr. Res., 338 (2003) 1981-1990.

[15] K. Sheth, Alexande.Jk, Biochim. Biophys. Acta, 148 (1967) 808-810.

[16] H.G. Tran, T. Desmet, K. Saerens, H. Waegeman, S. Vandekerckhove, M. D'hooghe, I. Van Bogaert, W. Soetaert, Bioresour. Technol., 115 (2012) 84-87.

[17] Ambar K. Choudhury, M. Kitaoka, K. Hayashi, Eur. J. Org. Chem., 2003 (2003) 2462-2470.

[18] T. Kawaguchi, Y. Ikeuchi, N. Tsutsumi, A. Kan, J.-I. Sumitani, M. Arai, J. Ferment. Bioeng., 85 (1998) 144-149.

[19] K. Hamura, W. Saburi, H. Matsui, H. Mori, Carbohydr. Res., 379 (2013) 21-25.

[20] M. Kitaoka, T. Sasaki, H. Taniguchi, J. Biochem., 112 (1992) 40-44.

[21] M. Kitaoka, H. Taniguchi, K. Hayashi, J. Appl. Glycosci., 49 (2002) 221-227.

[22] T. Sasaki, T. Tanaka, S. Nakagawa, K. Kainuma, Biochem. J., 209 (1983) 803-807.

[23] C. Sih, J. , in, Montana State College, 1955.

[24] C.J. Sih, R.H. McBee, Proc. Montana Acad. Sci., 15 (1955) 21-22.

[25] M. Arai, K. Tanaka, T. Kawaguchi, J. Ferment. Bioeng., 77 (1994) 239-242.

[26] M. Hiraishi, K. Igarashi, S. Kimura, M. Wada, M. Kitaoka, M. Samejima, Carbohydr. Res., 344 (2009) 2468-2473.

[27] M. Krishnareddy, Y.-K. Kim, M. Kitaoka, Y. Mori, K. Hayashi, J. Appl. Glycosci., 49 (2002) 1-8.

[28] D.M. Petrović, I. Kok, A.J.J. Woortman, J. Ćirić, K. Loos, Anal. Chem., 87 (2015) 9639-9646.

[29] E. Krissinel, K. Henrick, Acta Crystallogr., Sect. D: Biol. Crystallogr., 60 (2004) 2256-2268.

[30] X. Robert, P. Gouet, Nucleic Acids Res., 42 (2014) 320-324.
